# Methylation analysis of *Gasdermin E* shows great promise as a biomarker for colorectal cancer

**DOI:** 10.1002/cam4.2103

**Published:** 2019-04-16

**Authors:** Joe Ibrahim, Ken Op de Beeck, Erik Fransen, Lieselot Croes, Matthias Beyens, Arvid Suls, Wim Vanden Berghe, Marc Peeters, Guy Van Camp

**Affiliations:** ^1^ Centre of Medical Genetics University of Antwerp and Antwerp University Hospital Edegem Belgium; ^2^ Centre for Oncological Research University of Antwerp and Antwerp University Hospital Edegem Belgium; ^3^ StatUa Centre for Statistics University of Antwerp Antwerp Belgium; ^4^ Laboratory of Protein Chemistry, Proteomics and Epigenetic Signaling, Department of Biomedical Sciences University of Antwerp Antwerp Belgium; ^5^ Department of Medical Oncology Antwerp University Hospital Edegem Belgium

**Keywords:** Biomarker, Colorectal cancer, Diagnosis, DNA methylation, *GSDME*

## Abstract

In addition to its implication in hereditary hearing loss, the *Gasdermin E* (*GSDME*) gene is also a tumor suppressor involved in cancer progression through programmed cell death. *GSDME* epigenetic silencing through methylation has been shown in some cancer types, but studies are yet to fully explore its diagnostic/prognostic potential in colorectal cancer on a large‐scale. We used public data from The Cancer Genome Atlas (TCGA) to investigate differences in *GSDME* methylation and expression between colorectal cancer and normal colorectal tissue, and between left‐ and right‐sided colorectal cancers in 432 samples. We also explored *GSDME*'s diagnostic capacity as a biomarker for colorectal cancer. We observed differential methylation in all 22 *GSDME* CpGs between tumor and normal tissues, and in 18 CpGs between the left‐ and right‐sided groups. In the cancer tissue, putative promoter probes were hypermethylated and gene body probes were hypomethylated, while this pattern was inversed in normal tissues. Both putative promoter and gene body CpGs correlated well together but formed distinct methylation patterns with the putative promoter exhibiting the most pronounced methylation differences between tumor and normal tissues. Clinicopathological parameters, excluding age, did not show any effect on CpG methylation. Although the methylation of 5 distinct probes was a good predictor of gene expression, we could not identify an association between *GSDME* methylation and expression in general. Survival analysis showed no association between *GSDME* methylation and expression on 5‐year patient survival. Through logistic regression, we identified a combination of 2 CpGs, that can discriminate between cancer and normal tissue with high accuracy (AUC = 0.95) irrespective of age and tumor stage. We also validated our model in 3 external methylation datasets, from the Gene Expression Omnibus database, and similar results were reached. Our results suggest that *GSDME* is a promising biomarker for the detection of colorectal cancer.

## 1 INTRODUCTION

Colorectal cancer is the third most common cancer worldwide with 746 000 cases in men, 614 000 cases in women, and 694 000 deaths globally in 2012.^1^ Although incidence and mortality rates have been settling in several developed countries, the universal burden of colorectal cancer is projected to increase by more than 2.2 million cases and 1.1 million deaths by the year 2030.^1^ Its pathogenesis originates from epithelial cells lining the colon or rectum, which accumulate mutations in key cell signaling pathways such as Wnt signaling and, most commonly, in the APC tumor suppressor gene.^2^ Histologically, classical adenocarcinomas account for the majority of cases.^3^ More recently, varying disease characteristics have been described between left‐ and right‐sided colorectal tumors.^4^ Epigenetic changes in cancer, namely DNA methylation, have attracted great attention, especially with the availability of high‐throughput profiling methods. In colorectal cancer, normal methylation patterns are drastically reshaped and are characterized by widespread hypomethylation blocks.^5^, ^6^ Hypermethylation has also been observed in specific CpG islands and contributes to tumor initiation and progression.^7^, ^8^ Studies have already emphasized the substantial capacity of DNA methylation as biomarker for the early diagnosis of cancer.^9^, ^10^

The *Gasdermin E (GSDME) *gene was originally identified in our lab in 1998. Where gain of function mutations in the gene led to an autosomal dominant form of hearing loss. At the time of identification, its relation to other gasdermins had not yet been established, and the gene was initially named *Deafness Autosomal Dominant 5* *(DFNA5)*,^11^ only recently was it renamed *GSDME*. In addition to its role in hearing loss, *GSDME* is also a tumor suppressor gene capable of inducing programmed cell death as a result of a caspase‐3 cleavage.^12^ Given its role in cancer, epigenetic silencing through methylation has been shown in 52% and 65% of primary gastric tumors^13^ and colorectal adenocarcinomas^14^, ^15^ respectively. However, these studies were performed on a small number of tumors and only included a few CpGs from the putative promoter. More recently, *GSDME* putative promoter methylation has shown its potential as a biomarker for breast cancer.^16^, ^17^

To our knowledge, published large‐scale studies are yet to thoroughly evaluate the potential of *GSDME* methylation and expression as markers in colorectal cancer. We postulate that the methylation of *GSDME* could serve as a worthy biomarker for the detection of colorectal adenocarcinomas. This study aimed to analyze *GSDME* methylation and expression in the largest colorectal cancer patient dataset to date (N = 432) using publicly available data from The Cancer Genome Atlas (TCGA) in order to assess its diagnostic potential in colorectal cancer.

## 2 MATERIALS AND METHODS

### 2.1 Datasets and study population

The analyses presented in this manuscript were carried out on TCGA (colon and rectum adenocarcinoma) datasets that were downloaded from the GDC data portal website (https://portal.gdc.cancer.gov/) using an in‐house developed Python script. The script automates the querying of TCGA in order to easily and quickly download the data. TCGA stores patient sample data under unique barcodes following a specific layout; these are used to access biological and clinical data in the database. First, all patient barcodes available for colorectal cancer were downloaded via the website. API URLs were generated using the downloaded barcodes in order to query the matching TCGA level 3 methylation 450k Illumina platform data, the RNAseq V2 gene expression data, and the Agilent 244K microarray expression data. Subsequently, the methylation and gene expression data was downloaded for each barcode (patient) and stored in separated JSON formatted files. The individual JSON files were then merged per data type (methylation, RNAseq expression and microarray expression), through Python's dictionary functionality. This resulted in 3 data matrices, where sample data points (values: beta‐value or normalized counts) were column‐wise concatenated using the row name features (keys: methylation probe names or official gene symbols, 450K methylation or RNAseq V2 data respectively). The end result is a large table with probes row‐wise and samples column‐wise. The same principle was applied for downloading biospecimen and clinical data files. The biospecimen in the TCGA datasets were flash frozen/formalin‐fixed paraffin‐embedded, resection tissue samples, containing a minimum of 60% tumor nuclei and derived from primary, untreated colorectal tumor tissue. Using the in‐house script, methylation (level 3) data were obtained from the portal for all 22 *GSDME *CpGs.

To determine the probes' genomic location on the *GSDME* gene (gene body vs. putative gene promoter), we started from the most abundant *GSDME* RNAseq transcript (NM_004403) as the expression of the others was negligible. Additionally, NM_004403 is the only fully functional transcript of *GSDME*, coding for the full‐length protein of 496 amino acids. We annotated our transcript based on the GRCh37 Homo sapiens assembly which is also used by TCGA. To define the promoter and gene body regions of *GSDME* we used “Ensembl‐Regulatory Build of *GSDME* gene” where the promoter region is indicated based on data of the ENCODE project (promoter: 24795602‐24798199 and core promoter: 24797601‐24796400). We considered the core promoter together with the flanking regions (24795602‐ 24798199) as the putative promoter of *GSDME*. Based on this annotation, 6 CpGs (CpG1‐CpG6) are located in the gene body which extends from exon 2 until exon 10, 15 (CpG7‐CpG21) are located in the putative gene promoter which lies upstream of exon 2, while the last one (CpG22) is located in the upstream region, the details of which are described in Table 1.^18^ In this study however, we have considered CpG21 also a part of the upstream promoter region because its methylation pattern is clearly different from the other promoter CpGs and due to its very close proximity to the border of the *GSDME* promoter flanking region (24bp). Methylation is reported as β‐value, which is the ratio of the methylated probe intensity over the sum of methylated and unmethylated probe intensities, ranging from 0 to 1. These values were obtained by TCGA using the Illumina Infinium HumanMethylation450 BeadChip microarrays (Illumina Inc, San Diego, California). RNA sequencing (RNAseq) and microarray expression datasets were obtained in a similar fashion. RNAseq expression values in TCGA were acquired using the IlluminaHiSeq platform (Illumina, San Diego, California), and the respective transcript abundances were quantified using the Expectation Maximization algorithm. The expression values are reported as log2 transformed value and the highest predicted transcript for *GSDME* in RNAseq was the most abundant (NM_004403), while the expression of the other transcripts was negligible. Microarray expression values were obtained in TCGA using the Agilent 244K Custom Gene Expression G4502A‐07 microarrays (Agilent, Santa Clara, California) that contain 2 probes for *GSDME* (A_23_P82448 [36.3:chr.7:24705001‐24705060] and A_23_P82449 [36.3:chr.7:24705092‐24705151]), covering the 3 most abundant *GSDME* transcripts (NM_004403, NM_001127454.1, NM_001127453.1). Transcript NM_004403.2 was the most abundant, while the expression of the other transcripts was negligible and hence could not be included in the study. All microarray expression values are expressed as log2 transformed fold changes relative to the Universal Human Reference RNA (Stratagene).

**Table 1:** Table showing a simplified reference to the Illumina Infinium HumanMethylation450 probes, along with their genomic locations

Probe abbreviation	Probe name	Genomic coordinate	Location
CpG1	CpG17790129	24738572	Gene body
CpG2	CpG14205998	24748668	Gene body
CpG3	CpG04317854	24762562	Gene body
CpG4	CpG12922093	24767644	Gene body
CpG5	CpG17569154	24781545	Gene body
CpG6	CpG19260663	24791121	Gene body
CpG7	CpG09333471	24796355	Putative promoter
CpG8	CpG00473134	24796494	Putative promoter
CpG9	CpG03995857	24796553	Putative promoter
CpG10	CpG07320646	24796981	Putative promoter
CpG11	CpG07293520	24797192	Putative promoter
CpG12	CpG04770504	24797363	Putative promoter
CpG13	CpG24805239	24797486	Putative promoter
CpG14	CpG01733570	24797656	Putative promoter
CpG15	CpG25723149	24797680	Putative promoter
CpG16	CpG22804000	24797691	Putative promoter
CpG17	CpG07504598	24797786	Putative promoter
CpG18	CpG15037663	24797835	Putative promoter
CpG19	CpG19706795	24797839	Putative promoter
CpG20	CpG20764575	24797884	Putative promoter
CpG21	CpG06301139	24798175	Upstream region
CpG22	CpG26712096	24798855	Upstream region

**Figure 1:**
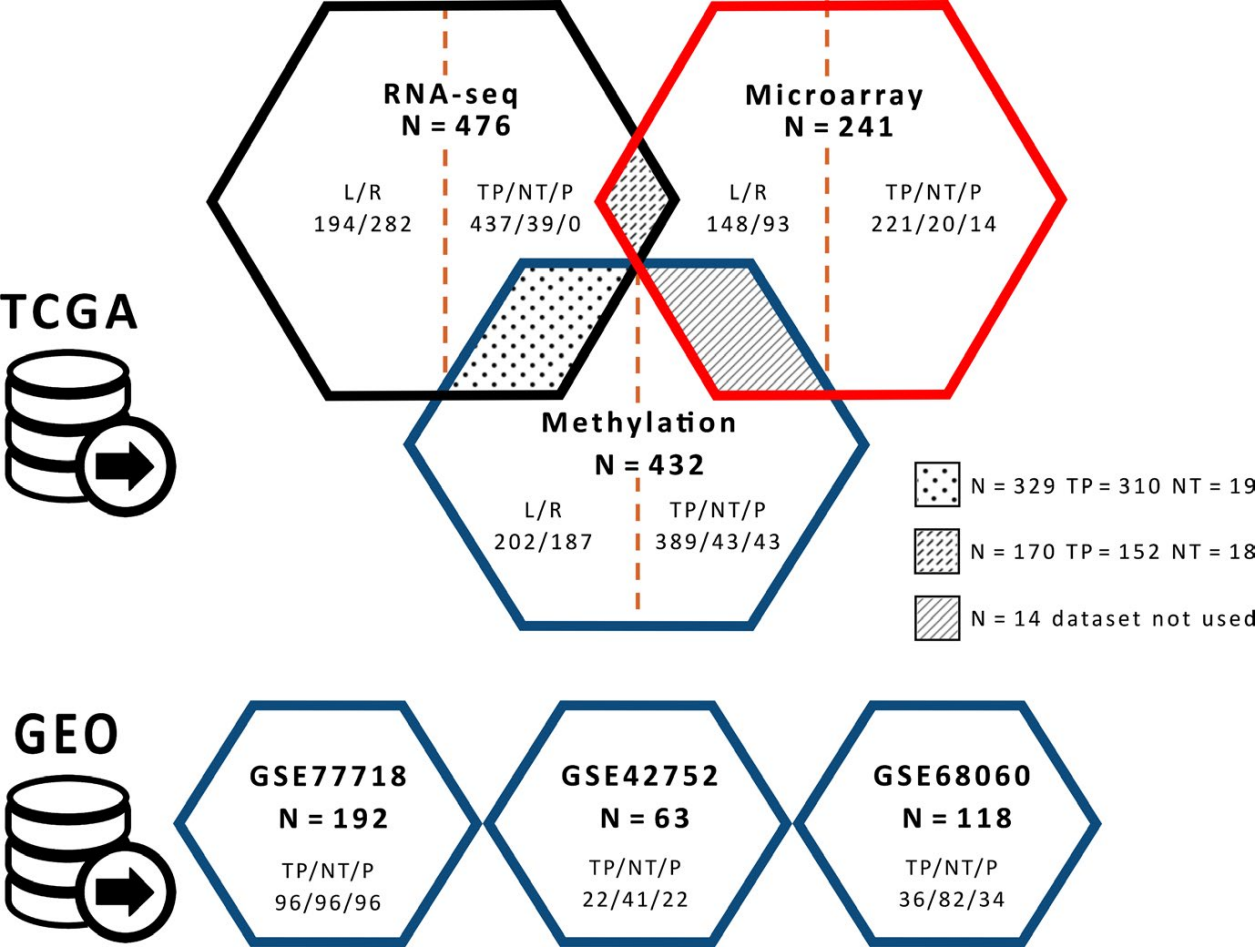
The different datasets used for hypothesis testing and result validation. Methylation and expression (RNAseq and microarray) datasets were obtained from TCGA, whereas additional methylation data were obtained from GEO for biomarker validation. TP: primary tumor, NT: normal tissue, P: paired samples (normal and tumor tissue from same individual), L: left‐sided CRC, R: right‐sided CRC

Primary tumor samples for which clinical data was available were then split into 2 categories: “left‐sided” and “right‐sided,” based on the anatomical location of the neoplasm, with the splenic flexure acting as the demarcation line between the 2 categories. Inherently, samples taken from the caecum, ascending colon, hepatic flexure and transverse colon were part of the right‐sided category, while samples from the splenic flexure, descending colon, sigmoid colon, rectosigmoid junction, and rectum comprised the left‐sided category. This categorization is based on the pragmatic split between the embryological origins of the colorectal tissue such that the right part of the colon originates from the midgut, while the left part is derived from the hindgut.^19^ After data filtering and classification, several final datasets were available for the downstream analyses. Information about these datasets is found in Figure 1 and Supplement Table S1.

### 2.2 Statistical analyses

We designated the following clinicopathological parameters from the TCGA clinical patient data files with which to carry out association analyses: age at diagnosis, gender, pathological tumor stage (I‐IV), anatomic neoplasm subdivision (left‐sided or right‐sided), and presence of colon polyps at procurement (Table 1). The *GSDME *sequence regions, methylation probe locations and chromatin states were explored using the UCSC genome browser.^20^ The statistical software R (version 3.4.1)^21^ was used to carry out all the statistical analyses. All reported p‐values are 2‐sided, and those less than or equal to 0.05 were considered statistically significant. To account for the nonindependence between measurements from the same individuals, a linear mixed model was fitted and included a random effect for sample barcodes. The significance of the fixed effects was then tested via the *F*‐test with a Kenward‐Roger correction for the number of degrees of freedom. Differences between groups were assessed through *t *tests and linear regression models, while association between expression and CpG methylation was tested using Spearman's correlation and through linear regression models. In all regression models age was accounted for as a covariate, but it was excluded from the final model if its effect on the outcome was not significant.

5‐year overall survival analysis was carried out by fitting Cox proportional hazard models to the methylation and expression datasets and including age as covariate. Additionally, stratified Cox models with separate baseline hazards for the 4 tumor stages were fitted. Censoring was carried out for individuals who died after the 5 years (1826 days) mark of the analysis and their respective follow‐up time was set to 1826 days. Quantile‐quantile plots showing the distribution of the 22 observed p‐values as compared to the uniform distribution, which is expected in the absence of any true association signal, were generated.

To assess the viability of *GSDME* methylation and expression as a biomarker for colorectal cancer, binary logistic regression was fitted to predict tissue type (normal/tumor) based on methylation and expression values with age as covariate. Stepwise multiple regression analysis was carried out to determine the best combination of the 22 CpGs. The final model was chosen based on the best Akaike information criterion (AIC) values with the lowest number of predictors possible. The accuracy of the model predictions was assessed by plotting receiver operating characteristic (ROC) curves. A 10‐fold cross validation of these results was then performed. Moreover, 3 additional Illumina 450K CpG methylation datasets were downloaded from the Gene Expression Omnibus (GEO) database (https://www.ncbi.nlm.nih.gov/geo/) (GEO accession numbers GSE77718, GSE42752, and GSE68060), and were used for the subsequent external validation (Figure 1). The final model was refit on each of the external datasets and the AUC was recalculated for the new predictions. The same methodology was also applied to RNAseq and microarray datasets to determine their predictive potential. A list of the R packages used in the analysis can be found on the last page of the Supplementary Material.

## 3 RESULTS

### 3.1 *GSDME* methylation and expression in primary untreated colorectal adenocarcinomas and histologically normal colorectal tissue

**Figure 2:**
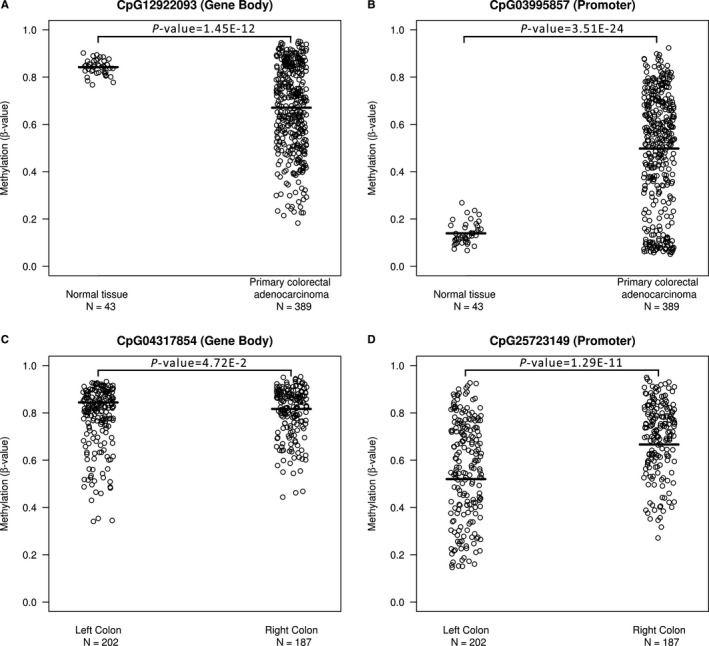
*GSDME* methylation differences between representative CpGs in the different sample groups. (A, B) The 2 presented CpGs exhibit the most significant differences in methylation levels between normal tissues (N = 43) and colorectal adenocarcinomas (N = 389). The lines indicate the mean *GSDME* methylation for each group; for CpG03995857(CpG10), the mean methylation is 0.14 (95% CI: 0.05, 0.23) in the normal tissue and 0.50 (95% CI: 0.03, 0.97) in the tumor tissue, while for CpG12922093(CpG4) these values are at 0.67 (95% CI: 0.31, 1.03) and 0.84 (95% CI: 0.78, 0.91) respectively. (C, D) CpG25723149(CpG15) is representative of *GSDME* promoter CpGs, where significant differences in methylation levels between left‐sided (N = 202) and right‐sided (N = 187) adenocarcinomas were observed in 15 out of 16 CpGs located in the putative gene promoter. For CpG25723149(CpG15), the mean methylation is 0.57 (95% CI: 0.15, 0.98) in the left colon and 0.70 (95% CI: 0.40, 0.99) in the right colon, while for CpG04317854(CpG3) these values are at 0.78 (95% CI: 0.53, 1.03) and 0.80 (95% CI: 0.60, 1.01) respectively

Our results showed a significant methylation difference between primary tumor and normal colorectal tissue for all 22 CpGs in the nonpaired samples (*P* = 3.51E‐24 to 3.94E‐2) and in 19 of 22 CpGs in the paired samples (*P* = 1.65E‐16 to 2.53E‐2) (Supplement Tables S2 and S3). For the significantly different CpGs located in the gene body, methylation levels in the normal tissue were higher than those in the tumor tissue, while the opposite holds true for CpGs located in the putative promoter region. The pattern switched again with 2 CpGs (CpG21 and CpG22), located upstream of the putative gene promoter region; these again showed increased methylation in the normal tissue as opposed to the tumor tissue (Figure 2A,B and Supplement Figure S1).

Two sources of *GSDME* expression were examined: RNAseq and microarray. The mean RNAseq expression for the normal tissues (5.80 95% CI: 3.31, 8.29) was slightly higher than that for the tumor tissues (5.45 95% CI: 2.68, 8.22), but these differences were not significant neither for the paired nor for the un‐paired samples. The same held true for microarray data where no significant differences were observed between the normal and tumor tissues (means of −3.18, 95% CI: −5.89, −1.38 and −0.46, 95% CI: −4.79, −1.38 respectively). Additionally, we explored the correlation between the 2 sources of *GSDME* expression data in samples for which both microarray and RNAseq *GSDME* expression values were available. The 2 datasets were highly correlated with a Spearman's coefficient of 0.89 for the whole datasets, 0.85 and 0.84 for the tumor tissues and normal tissues respectively, and 0.88 and 0.86 for the left‐sided and right sided groups respectively, all of which having a *P* < 2.2e‐16. With respect to the mentioned clinicopathological parameters, only age had a significant effect on the methylation of 14 out of 22 probes. These were CpGs 7‐18, 20 and 22 (Supplement Table S7). The calculated regression slopes were very close to zero (0.0002‐0.005) and as such the positive effect of age on probe methylation was somewhat minor; this is further elucidated by their weak Pearson's correlation coefficients (Supplement Table S7). Both the analysis of variance (ANOVA) and Tuckey tests resulted in nonsignificant *P*‐values, indicating that there is no significant difference in expression levels (microarray) between the different disease stages and that the observed variance is expression levels is due to intra‐stage differences.

### 3.2 *GSDME* methylation and expression in left‐sided and right‐sided colorectal adenocarcinomas

With respect to left‐sided and right‐sided colorectal adenocarcinomas, our investigation showed a significant difference in methylation levels between the subgroups for 18 out of 22 CpGs (*P* = 1.66E‐13 to 4.71E‐2) (Supplement Table S4). Interestingly, most significant differences were observed in the putative promoter region (CpG6‐22), whereas only 2 CpGs in the gene body were significantly different in methylation between the 2 groups (CpG1, *P* = 4.21E‐2 and CpG3, *P* = 4.71E‐2) (Supplement Table S4). For the significant CpGs, the methylation levels in the left‐sided subgroup were consistently lower than those in the right‐sided group and followed the general trend of putative promoter CpGs in the normal colorectal tissue (Figure 2C,D and Supplement Table S4). No significant differences in *GSDME *expression between the 2 groups were found. The correlation between methylation and expression in the left‐sided subgroup was 0.86 while in the right‐sided subgroup it was 0.84.

### 3.3 *GSDME* methylation and genomic location

**Figure 3:**
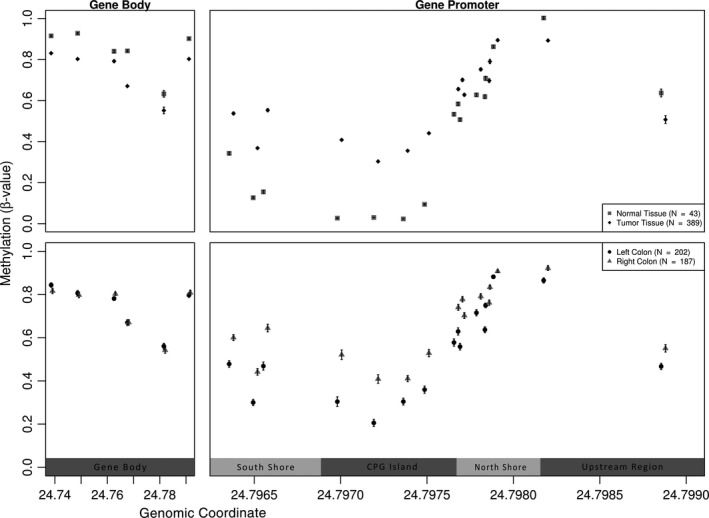
Physical map of the 22 CpGs in *GSDME*, correlating the chromosomal location with the average methylation values. The upper panel corresponds to the tumor versus normal tissues, while the lower panel corresponds to the different anatomical subgroups (left‐ and right‐sided). Error bars indicate the standard error of the mean. A clear trend can be observed in mean methylation values; normal samples are higher methylated in the gene body as compared to tumor samples while the opposite occurs for CpGs in the promoter region. The last 2 CpGs, located upstream of the putative gene promoter region, show a methylation pattern similar to intragenic CpGs. In the anatomical subgroups, differential methylation is found only in promoter CpGs, with an increased methylation observed in the right‐sided group as opposed to the left‐sided

After plotting the average *GSDME* methylation per CpG versus the respective physical map position on chromosome 7 (GRCh37), a clear trend in methylation was further elucidated (Figure 3). Methylation levels of the first 6 CpGs, located in the gene body, are higher in normal colorectal tissue as compared to tumor tissue. Conversely, the 14 following CpGs, located in the putative promoter region displayed a consistently lower methylation in the normal tissue as compared to tumor tissue. The inverse of this methylation pattern was seen for the last 2 CpGs, which are located upstream of the putative gene promoter region. As for the left‐sided and right‐sided groups, no difference can be seen in the methylation of gene body CpGs, in the putative promoter region the left‐sided group shows lower methylation compared to its counterpart (Figure 3).

**Figure 4:**
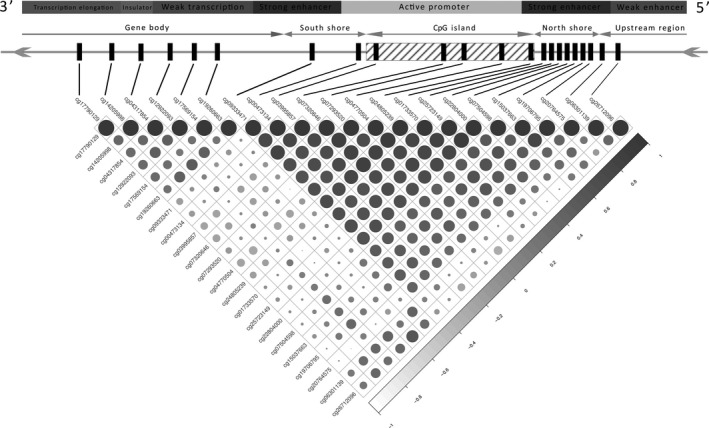
Correlation matrix of the methylation β‐values in the 22 CpGs of *GSDME* with genomic features overlay exhibiting a bloc‐like distribution. Correlation coefficients are indicated by circle color and size. All correlation coefficients had a *P *> 0.05. Two distinct clusters can be seen based on the correlation coefficients of the methylation values; promoter region CpGs form the biggest cluster (14 out of 22) while gene body CpGs for the smaller cluster, CpG21 and CpG22 cluster together and follow closely the pattern of the intragenic CpGs. On average methylation correlation in the putative promoter is stronger than that in the gene body, while the 2 regions do not correlate as well together. CpGs in the south and north shores comprise a strong enhancer region in the gene, whereas intragenic CpGs are located in region of relatively weak transcription

A correlation matrix for the methylation values of all 22 CpGs to investigate the association between the methylation of different regions in the *GSDME *gene, showed a block‐like clustering comprising a smaller cluster made up of the 6 CpGs located in the gene body, and a larger cluster made up of the remaining 14 CpGs located in the putative gene promoter region (Figure 4). Additionally, the last 2 CpGs located upstream of the putative gene promoter region clustered together and had a pattern similar to the gene body cluster. In these clusters, the larger CpG group, pertaining to probes in the putative promoter region, had the largest positive pairwise correlation coefficients whereas the smaller group had lower positive coefficients, all of which having significant *P *< 0.05 (Figure 4). Moreover, an accumulation of methylation was observed in the promoter region of tumor tissues with a significant 32% increase over the normal tissues. When excluding CpGs 21 and 22, which are thought to be upstream of the putative promoter region and clearly follow the methylation patterns of gene body CpGs (Figure 4), a 43% increase in methylation is observed. With respect to the gene body, a 13% decrease in methylation is observed in the tumor tissues as opposed to the normal. CpG islands are normally larger than 200 bp in length with a GC content above 50%. Shore regions are located up to 2 kilo base pairs upstream or downstream from the CpG island, while shelfs are regions 2 to 4 kilo base pairs away from the island.^22^ Based on the UCSC genome browser, a 946 bp CpG island was found to be part of the putative promoter, flanked by 2 enhancer regions (Figures 3 and 4). Moreover, high DNAse I activity is reported around the putative promoter region along with binding sites for E2F1 and PolR2A transcription factors.

### 3.4 Association between *GSDME* methylation and expression

**Figure 5:**
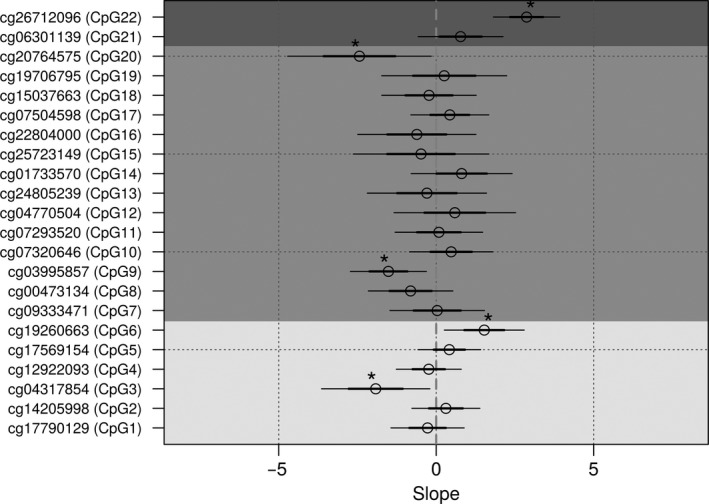
Regression plot for probe methylation as a predictor for gene expression. Thick lines indicate ±1 standard error, thin lines indicate ±2 standard error, while * indicates probes with significant *P*‐values (<0.05) Light shading represents intragenic CpGs, dark shading represents putative promoter CpGs, while the darkest shading represents CpGs upstream of the putative promoter region

We calculated the Spearman correlation coefficient to study the association between *GSDME* methylation and expression in samples for which both methylation data and expression data were available (RNAseq dataset), but none of the calculated correlation coefficients were strong (Supplement Table S5). Regression analysis over the whole dataset resulted in significant *P*‐values for CpGs 3, 6, 9, 20, and 22, this association however, was very weak indicated by the small exploratory variable slopes (Figure 5 and Supplement Table S5). Regression analysis on the grouped samples, showed that for the tumor samples about 40% of the variance in *GSDME* expression was attributable to variance in *GSDME* methylation (*R* ^2^ = 0.38, model *P* = 2.20E‐16). Five CpGs (CpG3, CpG6, CpG9, CpG20, CpG22) showed significant association between methylation *P*‐value and RNAseq expression. In the normal samples, a regression model could be fit, explaining around 60% of the variance in expression (*R* ^2^ = 0.63, model *P* = 1.10E‐2). However, only one CpG (CpG20 *P* = 3.50E‐2) showed a significant association with *GSDME* expression (Supplement Table S6). In the anatomical subgroups, around 40% of the variance could also be explained by the CpGs included in the models. In the left‐sided group the methylation of only 2 CpGs (CpG9, CpG22) showed a significant association with *GSDME* expression, while in the right‐sided group 4 CpGs (CpG6, CpG9, CpG20, CpG22) were significantly associated (Supplement Table S6). Overall, the regression analysis showed a heterogeneity in the effects of CpG methylation on expression. The coefficients were spread between positive and negative values with most of them clustering around zero, indicating minor effects between the variables (Figure 5). The results in both datasets are relatively disparate and hence the contribution of *GSDME* methylation to expression levels is still inconclusive, with no consistent association between the 2.

### 3.5 Associations between *GSDME* methylation or expression and 5‐year overall survival

The association between survival and methylation or expression was studied using Cox proportional‐hazard models in patients for which follow‐up data were available (N = 260). For the complete adenocarcinoma dataset, no significant association between methylation and 5‐year survival could be found. For the left‐sided and right‐sided subgroups, a significant association was found only for 1 CpG (CpG22 *P* = 1.60E‐02) and for 2 CpGs (CpG4 *P* = 1.51E‐2, CpG21 *P* = 3.13E‐2), respectively. By comparing the distribution of the *P*‐values to the expected distribution under the null hypothesis of no association, no enrichment in low *P*‐values was observed and hence CpG methylation does not seem to be a strong predictor of 5‐year survival (Supplement Figures S2, S3 and Supplement Table S8). We repeated the same analysis for both RNAseq and microarray expression data, but again no clear association could be deduced. It is noteworthy that in all hazard proportion models, only age had a significant influence on survival (Supplement Figures S2, S3 and Supplement Table S8).

### 3.6 *GSDME* methylation and expression as potential detection biomarker for colorectal adenocarcinomas

**Figure 6:**
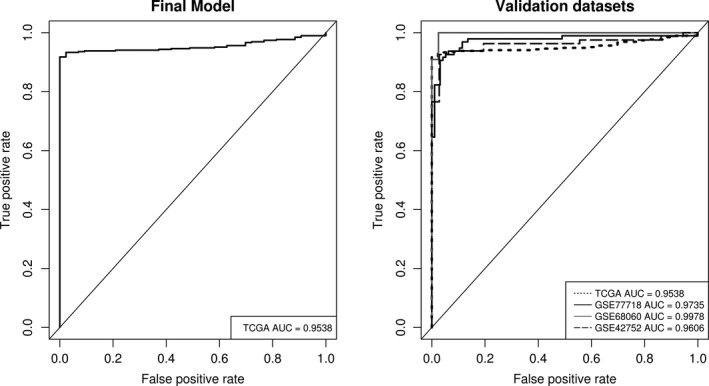
*GSDME* CpG methylation as biomarker for colorectal adenocarcinomas. The left panel shows the ROC curve of the final prediction model taking one CpG in the gene body (CpG4) and one CpG in the gene promoter (CpG17) as predictors and accounting for age. Sensitivity and specificity at various cutoff values for the TCGA dataset are plotted resulting in a 0.95 (95% CI: 0.95, 0.98) AUC. At a set cutoff value of 0.72, sensitivity and specificity were at 93.3% and 93.7% respectively while overall model accuracy was 97.6%. The right panel shows ROC curves for the subsequent validation of the model by 3 external datasets. The AUCs for the external datasets were very similar to that of the original data thus confirming the diagnostic value of the model and its generalizability over other datasets. The diagonal line represents the line of no discrimination between tumor and normal colorectal tissues

In a logistic regression framework, we explored all combinations of the 22 CpGs that would yield discriminatory power to distinguish between tumor and non‐tumor tissue states. Six CpGs had good predictive value in our models (CpG12, CpG14, CpG4, CpG17, CpG15, CpG2). In general, models with 2 CpGs led to a better prediction than those with only one. Their AUC values were in the range of 0.72‐0.97 and 0.71‐0.87, respectively (Figure 6, Table 1, and Supplement Figure S4). To analyze if the relation between CpG methylation and disease status (or tissue type) is homogeneous across tumor stages, we fitted logistic regression models. Tissue type was entered as dependent variable, and independent variables included CpG methylation, stage and the interaction between methylation and stage. The significance of this latter term (effect/interaction between methylation and diseases stage) tests the null hypothesis of homogeneity of the marker across the stages: in case the *P*‐value of the interaction is significant, then the association between the CpG methylation and tissue type is not uniform across stages, in other terms, that CpG methylation is significantly different depending on disease stage. The significance of the interaction term was tested using a likelihood ratio test, comparing the fit of the model with both main effects and their interaction term, against the model with only the main effects of methylation and stage. None of the stages or interaction terms showed a significant outcome on tissue type prediction and hence methylation was not significantly altered by stage, reinforcing the marker's homogeneity across disease stages (Supplement Table S9).

For our final prediction model, CpG 12 located in the putative promoter region and CpG4 located in the gene body were chosen as predictors, resulting in a 0.95 (95% CI: 0.95‐0.98) AUC value. A 10‐fold cross‐validation resulted in an AUC value of 0.95 (95% CI: 0.93‐0.97, SE = 0.01). Sensitivities and specificities at the different cutoff values for the predicted probabilities are shown by means of an ROC plot (Figure 6). At a cutoff value of 0.72, a sensitivity of 93.3% and a specificity of 93.7% for detection of colorectal adenocarcinomas were reached without false positives, with an overall accuracy of 97.6%. As an external validation, we applied our trained model to 3 external CpG methylation datasets downloaded from the GEO database (Figure 1) using the same 2 CpGs as predictors. Sample tissue type was successfully predicted in all 3 datasets with AUC values comparable to that of the original TCGA dataset; GSE77718, GSE42752, and GSE68060 had AUCs of 0.97, 0.96 and 0.99 respectively. In all, the model exhibited a high predictive power and good generalizability across different datasets (Figure 6, Table 1).

We additionally investigated the potential of *GSDME* expression data as a biomarker. Using the same methodology, a ROC curve was constructed using RNAseq data for 453 tumor tissues and 41 normal tissues and microarray data for 221 tumor tissues and 20 normal tissues. The AUC values were 0.55 and 0.60 respectively, reflecting a low predictive power.

## 4 DISCUSSION

Due to the rise of cost‐effective genome‐wide profiling methods such as the Illumina Infinium HumanMethylation450 and the more recent MethylationEPIC beadchips, several studies have emerged linking aberrant DNA methylation to the onset and progression of colorectal tumors.^23^, ^24^ Originally discovered in our lab as a gene responsible for autosomal hearing loss, *GSDME* was also identified as a target of epigenetic silencing in several cancer types.^13^, ^14^, ^15^, ^16^, ^17^ In this analysis of TCGA methylation data, we provide evidence that *GSDME* DNA methylation is a promising biomarker for the diagnosis of colorectal cancer.

Differential methylation was observed across*GSDME* (Figure 2A,B and Supplement Figure S1, Supplement Tables S2 and S3). The putative promoter region of healthy tissues was hypomethylated as compared to the gene body, whereas the opposite was observed in tumor tissues. Our findings conform to the global genomic methylation paradigm of CpGs in cancer,^24^ with *GSDME*'s methylation patterns following those of other known tumor suppressor genes in colorectal cancer.^13^, ^14^, ^24^ The methylation status of probes in the putative promoter region were highly correlated, as was the case with those in the gene body region, but not between these 2 distinct regions, and a clear positional segregation between them was observed. On average, the largest and most significant differences in methylation were observed in putative promoter CpGs as compared to the gene body. This clear difference between the 2 regions is further illustrated through the bimodal clustering of methylation correlation coefficients in the matrix (Figure 4). CpGs 1‐6, which are located in the gene body are highly correlated together, as are CpGs 7‐19, which are located in the putative gene promoter. CpGs 21 and 22, which are thought to be located upstream of the putative promoter, correlate strongly with CpGs 1‐6, and follow their methylation patterns. The methylation levels of the 2 CpG clusters seems to act conversely; levels of putative promoter CpGs seemed to increase simultaneously in tumor tissues, while that of gene body CpGs decreased in a similar fashion. The opposite was true when considering normal tissues. To date the impact of CpGs located outside of promoter regions, such as intragenic CpGs, is yet to be fully understood as they do not seem to directly regulate gene expression. Nevertheless, a few studies have already hinted at their potential role in modulating alternative promoters 25 or in long‐range regulation.^26^ More recently, Neri et  al^27^, proposed intragenic DNA methylation as a safeguard against spurious transcription initiation. Its absence in cancerous cells could affect biological processes and favour neoplastic predisposition. Despite the scarcity of studies on *GSDME* methylation, our findings are still in agreement with those reported for colorectal^14^, ^15^ and breast cancer^16^, ^17^ albeit their analyses involved TaqMan‐MSP and pyrosequencing respectively, as opposed to the Illumina 450K beadchip data that was used here. Our results show that methylation alterations occur not only within the strict putative gene promoter but also in the mentioned shore regions (Figures 3, 4 and Supplement Tables S2 and S3), which is in agreement with the original findings of Irizarry et al that outlined the involvement of CpG island shores in colon cancer.^28^ The observed higher methylation in the gene body of normal cells is hypothesized to act as a protective strategy against unwanted intragenic transcription from highly active genes and the possible production of aberrant gene products. Such methylation patterns are hypothesized to protect the gene body from faulty RNA polymerase II entry and cryptic transcription initiation. Evidently, tumors are characterized by global DNA hypomethylation, especially in intragenic regions, a fact that hints at a functional epigenetic crosstalk between DNA methylation, RNA polymerase II and gene expression.^27^ Taken together, we believe that our findings are in line with the global CpG methylation paradigm of tumor suppressors in cancer.^29^ Clinical parameters did not have any significant outcome on methylation, with the exception of age, which showed a minor positive effect on 14 out of the 16 CpGs in the putative promoter region, for one of the CpGs upstream of that region a minor negative effect was observed (Supplement Table S7).

We investigated the association between *GSDME* methylation and expression through correlation tests and linear regression, but no significant correlation between them, for both tumor and normal tissues could be observed. Some association between methylation and expression was found in 5 distinct probes across the tested groups (Figure 5 and Supplement Tables S5 and S6), of which CpGs 20 and 22 recurred in 3 out of the 4 groups followed by CpG9 in 2 groups. The effect of methylation on expression seemed low and disparate, even for the significant probes, with the exception of CpG20, whose methylation showed a fivefold positive effect on RNAseq expression. Due to the discrepancy in the results, we could not establish a conclusive relationship between *GSDME* methylation and its expression, which is somewhat discordant with the general dogma of gene expression in cancer and in particular with the findings of Akino et al that showed a significant downregulation in colorectal cancer tissues as compared to normal tissue.^13^ Although a strictly linear relationship between *GSDME* methylation and expression is not anticipated, we would expect that increased methylation leads to decreased gene expression in cancer. The discrepancy in our results and in conflicting findings,^13^, ^30^ make it hard to clearly define the effect of *GSDME* methylation on its expression, particularly in light of recent studies suggesting that the relation between the 2 could be more complex.^31^ One conflicting factor could be miRNAs (mir‐3p and mir26b‐5p) that are known to modulate *GSDME* expression,^32^ and another could be copy number variations (CNVs) that play an important role in complex phenotypes such as cancer.^33^ Moreover, the location of CpG methylation (in or near the island) is crucial to its influence on gene expression such that CpGs located outside the islands have a bigger impact on gene expression than those located within.^34^

A novel finding of this study is the differential methylation between left‐sided and right sided tumors. Several studies have outlined the differences between the 2 groups, attributing higher incidence but better prognosis to left‐sided tumors versus right‐sided ones^4^; however, differential methylation patterns have not yet been widely considered. Our findings are in line with various communications that have reported a significant association between hypermethylated CpGs and right‐sided tumor locations.^35^, ^36^, ^37^ This same rationale could also apply to the worse disease progression and prognosis of the right‐sided group where we report higher *GSDME *methylation. *GSDME* could be one of several tumor suppressor genes that are differentially activated between left‐ and right‐sided colorectal cancers leading to differences in prognosis and survival. To date, there is no clear explanation for the observed methylation differences. Considering that diet type is one of the main risk factors of colorectal cancer,^38^ we speculate that the concentration gradient of substances or gut microbiota across different parts of the colon, might be one of the causes of such methylation patterns. Another could be the divergent embryological origins of the colon and their inherent cellular differences. Tumor location has a prognostic value in CRC as it provides information about the overall cancer outcome, independent of treatment received. Its predictive value on the other hand, provides information on the likelihood of response to a given therapy, and therefore helps to optimize treatment decisions. In addition to microsatellite instability, chromosome instability, invasive polymicrobial biofilms and gene mutations (*KRAS*, *BRAF*, *PIK3CA*, *NRAS,* and *TPS3*), the difference in *GSDME* methylation (amongst other genes) could reflect a different disease etiology and outcome, but to clarify this, more research is necessary.

With respect to colorectal cancer screening, several tests have been devised, including the fecal immunochemical test (FIT) and the fecal DNA test.^39^ Moreover, genes with aberrant methylation patterns have been reported as biomarkers, for example, the silencing of *VIM *through promoter methylation has been exploited clinically as a tumor determinant as part of the Cologuard kit.^40^ More recently, SEPT*9 *promoter hypermethylation was approved for use in colorectal cancer screening as part of 3 commercially available kits (Epi proColon, ColoVantage, and RealTime mS9).^41^ Despite their near perfect specificity and superior performance over FIT, such methods still compromise on sensitivity and some even show significant variability based on disease stage,^42^, ^43^, ^44^, ^45^, ^46^ hence the need for new and improved biomarkers remains. In our case, the identified CpGs performed well in tumor classification with both a high sensitivity and specificity. The external validation, confirms the model's validity and its generalizability over external cohorts and colorectal cancer tissue types (Figure 6, Table 1). Normally, the level of aberrant DNA methylation of genes is higher in later stages as compared to early stages in most cancers. This makes it difficult to identify genes that can be used in a diagnostic setting as their methylation status is highly dependent on disease progression and is not always detected in early stages where the need for diagnosis is paramount.^39^, ^41^ In *GSDME*'s case, our prediction model metrics were unaffected by age and disease stage (Supplement Table S9). This makes *GSDME* an excellent candidate gene for early diagnosis irrespective of CRC stage as its aberrant methylation status can be ubiquitously used for predictions across disease progression. Although most combinations of 2 probes yielded good prediction results (Supplement Figure S4), the final step‐wise binary logistic regression model included CpGs 2, 4, 12‐17 and 21. These probes had the highest contribution in accurately predicting tissue type based on methylation values. CpGs 4 and 12 yielded the best AUC, thus their importance in the biomarker context. Given the strong correlation between probes in the different regions, pairwise combinations using any of the mentioned probes still yielded a high prediction AUC. Overall, differences in methylation were more prominent in the putative promoter region as compared to the gene body, with CpG9 and CpG4 having the highest mean differences respectively, and more of the putative promoter probes contributing to the prediction model. Moreover, the largest differences in methylation were observed in CpGs 9, 10, 12, and 13, all of which exhibited the highest tissue prediction capacities in both the single and pairwise models (Supplement Table S10 and Supplement Figure S4). CpG13 exhibited the most variation across all our analyses and is possibly located within a region of high epigenetic activity. CpG12 is the most important clinically as it yielded the best ROC curve and had the biggest contribution to tissue type prediction.

Our findings regarding *GSDME *methylation can be translated into a noninvasive biomarker assay through digital‐droplet PCR (dd‐PCR) for example. To that end, the next step would be investigating *GSDME* methylation in collected tissue biopsies from a cohort of colorectal cancer patients and healthy individuals. This would then be followed by testing in circulating tumor DNA extracted from a similar cohort using blood‐based liquid biopsies. Evidently, such an assay would still need to undergo clinical trials and be compared to existing early detection kits before making its way to the clinic. To date, a handful of “proof of concept” studies have emerged, outlining the basis of such methods in breast and colorectal cancers.^47^, ^48^, ^49^ Given that colorectal cancer initially develops from colon polyps, and that epigenetic modifications are believed to be precursors of cancer formation,^5^ another step would be testing *GSDME *methylation in polyps to assess its potential as an early biomarker for colorectal cancer. Considering the models’ robustness and the low number of predictor probes needed, the answer might ultimately lie in the use of *GSDME* methylation in liquid biopsies as a sensitive, minimally‐invasive and cost‐effective detection method for colorectal cancer.

The exceptional in silico performance of the thoroughly identified CpG dinucleotides in a large patient cohort, makes this study a stepping stone towards developing a biomarker assay for the detection of colorectal cancer, in the context of liquid biopsy‐based dd‐PCR. With respect to left‐sided versus right‐sided colorectal cancer, the underlying causes of nonadherence and their effects on treatment outcome warrant further investigation. In this context, exploring the differences in methylation and expression between additional clinicopathological parameters, such as neoadjuvant therapy status or microsatellite instability would be beneficial to understanding their effects on tumor sidedness. One limitation of this study is the lack of clinically collected tissue methylation data; hence the next step would be the validation of in silico data using tissue biopsies.

## References

[1] Ferlay J , Soerjomataram I , Dikshit R , et al. Cancer incidence and mortality worldwide: Sources, methods and major patterns in GLOBOCAN 2012. Int J Cancer. 2015;136:E359‐E386. 25220842 10.1002/ijc.29210

[2] Kinzler KW , Vogelstein B . Lessons from hereditary colorectal cancer. Cell. 1996;87:159‐170. 8861899 10.1016/s0092-8674(00)81333-1

[3] Bosman FT , Carneiro F , Hruban RH , Theise ND . WHO Classification of Tumours of the Digestive System (4th edn). Lyon, France: International Agency for Research on Cancer; 2010.

[4] Boeckx N , Janssens K , Van Camp G , et al. The Predictive Value of Primary Tumor Location in Patients with Metastatic Colorectal Cancer: A Systematic Review. Crit Rev Oncol Hematol. 2018;121:1-10. 29279095 10.1016/j.critrevonc.2017.11.003

[5] Okugawa Y , Grady WM , Goel A . Epigenetic alterations in colorectal cancer: emerging biomarkers. Gastroenterology. 2015;149:1204‐1225.e12. 26216839 10.1053/j.gastro.2015.07.011 PMC4589488

[6] Timp W , Bravo HC , McDonald OG , et al. Large hypomethylated blocks as a universal defining epigenetic alteration in human solid tumors. Genome Med. BioMed Central. 2014;6:61. 25191524 10.1186/s13073-014-0061-y PMC4154522

[7] Luo Y , Wong CJ , Kaz AM , et al. Differences in DNA methylation signatures reveal multiple pathways of progression from adenoma to colorectal cancer. Gastroenterology. NIH Public Access. 2014;147:418–429.e8. 24793120 10.1053/j.gastro.2014.04.039 PMC4107146

[8] Naumov VA , Generozov EV , Zaharjevskaya NB , et al. Genome‐scale analysis of DNA methylation in colorectal cancer using Infinium HumanMethylation450 BeadChips. Epigenetics. Taylor & Francis. 2013;8:921‐934. 10.4161/epi.25577 PMC3883769 23867710

[9] Dong Y , Zhao H , Li H , Li X , Yang S . DNA methylation as an early diagnostic marker of cancer (Review). Biomed Rep. Spandidos Publications. 2014;2:326‐330. 24748968 10.3892/br.2014.237 PMC3990206

[10] Lam K , Pan K , Linnekamp JF , Medema JP , Kandimalla R . DNA methylation based biomarkers in colorectal cancer: a systematic review. Biochim Biophys Acta ‐ Rev Cancer. 2016;1866:106‐120. 10.1016/j.bbcan.2016.07.001 27385266

[11] Laer LV , Huizing EH , Verstreken M , et al. Nonsyndromic hearing impairment is associated with a mutation in DFNA5. Nat Genet. 1998;20:194‐197. 9771715 10.1038/2503

[12] Rogers C , Fernandes‐Alnemri T , Mayes L , et al. Cleavage of DFNA5 by caspase‐3 during apoptosis mediates progression to secondary necrotic/pyroptotic cell death. Nat Commun. Nature Publishing Group; 2017;8:14128. 10.1038/ncomms14128 PMC5216131 28045099

[13] Akino K , Toyota M , Suzuki H , et al. Identification of DFNA5 as a target of epigenetic inactivation in gastric cancer. Cancer Sci. 2006;98:88‐95. 10.1111/j.1349-7006.2006.00351.x PMC11158324 17083569

[14] Kim Ms , Chang X , Yamashita K , et al. Aberrant promoter methylation and tumor suppressive activity of the DFNA5 gene in colorectal carcinoma. Oncogene. 2008;27:3624‐3634. 18223688 10.1038/sj.onc.1211021

[15] Yokomizo K , Harada Y , Kijima K , et al. Methylation of the DFNA5 gene is frequently detected in colorectal cancer. Anticancer Res. 2012;32:1319‐1322. 22493364

[16] Croes L , de Beeck KO , Pauwels P , et al. DFNA5 promoter methylation a marker for breast tumorigenesis. Oncotarget. Impact Journals, LLC, 2017;8:31948‐31958. 28404884 10.18632/oncotarget.16654 PMC5458261

[17] Kim MS , Lebron C , Nagpal JK , et al. Methylation of the DFNA5 increases risk of lymph node metastasis in human breast cancer. Biochem Biophys Res Commun. 2008;370:38‐43. 18346456 10.1016/j.bbrc.2008.03.026 PMC3094717

[18] Op de Beeck K , Van Camp G , Thys S , et al. The DFNA5 gene, responsible for hearing loss and involved in cancer, encodes a novel apoptosis‐inducing protein. Eur J Hum Genet. 2011;19:965‐973. 21522185 10.1038/ejhg.2011.63 PMC3179363

[19] Price TJ , Beeke C , Ullah S , et al. Does the primary site of colorectal cancer impact outcomes for patients with metastatic disease? Cancer. 2015;121:830‐835. 25377235 10.1002/cncr.29129

[20] Kent JW , Sugnet CW , Furey TS , et al. The human genome browser at UCSC. Genome Res. Cold Spring Harbor Laboratory Press; 2002;12:996‐1006. 12045153 10.1101/gr.229102 PMC186604

[21] R Core Team . A Language and Environment for Statistical Computing. Vienna, Austria: R Foundation for Statistical Computing; 2017.

[22] Sandoval J , Heyn H , Moran S , et al. Validation of a DNA methylation microarray for 450,000 CpG sites in the human genome. Epigenetics. 2011;6:692‐702. 21593595 10.4161/epi.6.6.16196

[23] Durso DF , Bacalini MG , do Valle , et al. Aberrant methylation patterns in colorectal cancer: a meta‐analysis. Oncotarget. Impact Journals, LLC, 2017;8:12820‐12830. 28086223 10.18632/oncotarget.14590 PMC5355058

[24] Goel A , Boland CR . Epigenetics of colorectal cancer. Gastroenterology. NIH Public. Access. 2012;143:1442‐1460.e1. 23000599 10.1053/j.gastro.2012.09.032 PMC3611241

[25] Maunakea AK , Chepelev I , Cui K , Zhao K . Intragenic DNA methylation modulates alternative splicing by recruiting MeCP2 to promote exon recognition. Cell Res. 2013;23:1256‐1269. 23938295 10.1038/cr.2013.110 PMC3817542

[26] Kulis M , Queirós AC , Beekman R , Martín‐Subero JI . Intragenic DNA methylation in transcriptional regulation, normal differentiation and cancer. Biochim Biophys Acta ‐ Gene Regul Mech. Elsevier B.V. 2013;1829:1161‐1174. 10.1016/j.bbagrm.2013.08.001 23938249

[27] Neri F , Rapelli S , Krepelova A , et al. Intragenic DNA methylation prevents spurious transcription initiation. Nature. Nature Publishing Group. 2017;543:72‐77. 28225755 10.1038/nature21373

[28] Irizarry RA , Ladd‐Acosta C , Wen B , et al. The human colon cancer methylome shows similar hypo‐ and hypermethylation at conserved tissue‐specific CpG island shores. Nat Genet. NIH Public Access. 2009;41:178‐186. 19151715 10.1038/ng.298 PMC2729128

[29] Esteller M . Molecular Origins of Cancer Epigenetics in Cancer. N Engl J Med. 2008;358:1148‐1159. 18337604 10.1056/NEJMra072067

[30] Stoll G , Ma Y , Yang H , Kepp O , Zitvogel L , Kroemer G . Pro‐necrotic molecules impact local immunosurveillance in human breast cancer. Oncoimmunology. Taylor & Francis. 2017;6:e1299302. 28507808 10.1080/2162402X.2017.1299302 PMC5414877

[31] Long MD , Smiraglia DJ , Campbell MJ . The genomic impact of DNA CpG methylation on gene expression; relationships in prostate cancer. Biomolecules. 2017;7:1‐20. 10.3390/biom7010015 PMC5372727 28216563

[32] Chou CH , Chang NW , Shrestha S , et al. miRTarBase 2016: Updates to the experimentally validated miRNA‐target interactions database. Nucleic Acids Res. 2016;44:D239‐D247. 26590260 10.1093/nar/gkv1258 PMC4702890

[33] Vaquerizas JM , Kummerfeld SK , Teichmann SA , Luscombe NM . A census of human transcription factors: function, expression and evolution. Nat Rev Genet. 2009;10:252‐263. 19274049 10.1038/nrg2538

[34] Van Vlodrop I , Niessen H , Derks S , et al. Analysis of promoter CpG island hypermethylation in cancer: Location, location, location! Clin Cancer Res. 2011;17:4225‐4231. 21558408 10.1158/1078-0432.CCR-10-3394

[35] Lee MS , McGuffey EJ , Morris JS , et al. Association of CpG island methylator phenotype and EREG/AREG methylation and expression in colorectal cancer. Br J Cancer. 2016;114:1352‐1361. 27272216 10.1038/bjc.2016.87 PMC4984478

[36] Simons C , Hughes L , Smits Km , et al. A novel classification of colorectal tumors based on microsatellite instability, the CpG island methylator phenotype and chromosomal instability: implications for prognosis. Ann Oncol. 2013;24:2048‐2056. 23532114 10.1093/annonc/mdt076

[37] Zong L , Abe M , Ji J , Zhu W‐G , Yu D . Tracking the correlation between CpG island methylator phenotype and other molecular features and clinicopathological features in human colorectal cancers: a systematic review and meta‐analysis. Clin Transl Gastroenterol. 2016;7:e151. 26963001 10.1038/ctg.2016.14 PMC4822093

[38] Fleming M , Ravula S , Tatishchev SF , Wang HL . Colorectal carcinoma: Pathologic aspects. J Gastrointest Oncol. 2012;3:153‐173. 22943008 10.3978/j.issn.2078-6891.2012.030 PMC3418538

[39] Song L‐L , Li Y‐M . Current noninvasive tests for colorectal cancer screening: An overview of colorectal cancer screening tests. World J Gastrointest Oncol. 2016;8:793. 27895817 10.4251/wjgo.v8.i11.793 PMC5108981

[40] Li Y‐W , Kong F‐M , Zhou J‐P , Dong M . Aberrant promoter methylation of the vimentin gene may contribute to colorectal carcinogenesis: a meta‐analysis. Tumor Biol. Springer Netherlands, 2014:35:6783‐6790. 10.1007/s13277-014-1905-1 24729088

[41] Semaan A , van Ellen A , Meller S , et al. SEPT9 and SHOX2 DNA methylation status and its utility in the diagnosis of colonic adenomas and colorectal adenocarcinomas. Clin Epigenetics. 2016;8:100. 27660666 10.1186/s13148-016-0267-5 PMC5028994

[42] Shirahata A , Hibi K . Serum vimentin methylation as a potential marker for colorectal cancer. Anticancer Res. International Institute of Anticancer Research, 2014;34:4121‐4126. 25075038

[43] Han X , Wang J , Sun Y . Circulating tumor DNA as Biomarkers for cancer detection. Genom Proteom Bioinf. Beijing Institute of Genomics, Chinese Academy of Sciences and Genetics Society of China, 2017;15:59‐72. 10.1016/j.gpb.2016.12.004 PMC5414889 28392479

[44] Leygo C , Williams M , Jin HC , et al. DNA methylation as a noninvasive epigenetic biomarker for the detection of cancer. Dis Markers. Hindawi, 2017;2017:1‐13. 10.1155/2017/3726595 PMC5605861 29038612

[45] Chen CH , Yan SL , Yang TH , et al. The relationship between the methylated septin‐9 DNA blood test and stool occult blood test for diagnosing colorectal cancer in Taiwanese people. J Clin Lab Anal. 2017;31:e22013. 10.1002/jcla.22013 PMC6817212 27390118

[46] Mikeska T , Craig JM . DNA methylation biomarkers: Cancer and beyond. Genes (Basel). 2014;5:821‐864. 25229548 10.3390/genes5030821 PMC4198933

[47] Wittenberger T , Sleigh S , Reisel D , et al. DNA methylation markers for early detection of women’s cancer: promise and challenges. Epigenomics. 2014;6:311‐327. 25111485 10.2217/epi.14.20

[48] Warton K , Mahon KL , Samimi G . Methylated circulating tumor DNA in blood: Power in cancer prognosis and response. Endocr Relat Cancer. 2016;23:R157‐R171. 26764421 10.1530/ERC-15-0369 PMC4737995

[49] Garrigou S , Perkins G , Garlan F , et al. A study of hypermethylated circulating tumor DNA as a universal colorectal cancer biomarker. Clin Chem. 2016;62:1129‐1139. 27251038 10.1373/clinchem.2015.253609

