# Supplementary material for: Methylation analysis of Gasdermin E shows great promise as a biomarker for colorectal cancer
Source: Cancer Med. 2019 Apr 16;8(5):2133–45. doi: 10.1002/cam4.2103 (PMC6536921; doi:10.1002/cam4.2103)
Supplement: Supplementary file 1 [file CAM4-8-2133-s001.docx]

**Supplementary Table 1. Study population characteristics of the TCGA methylation and expression datasets.**

|  | Methylation Dataset | RNAseq Dataset | Microarray Dataset |
| --- | --- | --- | --- |
| Sample size | 432 | 476 | 241 |
| Sample type (tumour/normal) | 389/43 | 437/39 | 221/20 |
| Gender (m/f) | 231/201 | 249/227 | 121/120 |
| Mean age ± SD | 64.95 ± 13.08 | 67.16 ± 13.10 | 69.48 ± 11.84 |
| Neoadj. therapy (yes/no) | 1/431 | 4/472 | 3/238 |
| Colon polyps (yes/no) | 97/208* | 78/151* | 5/25* |
| Stage (I/II/III/IV) | 65/164/128/58* | 77/193/129/66* | 49/97/56/38* |
| Anatomic subdivision (left/right) | 202/187 | 194/282 | 148/93 |

SD = standard deviation, Neoadj. therapy = neoadjuvant therapy, *field with missing values

**Supplementary Table 2. Differences in *GSDME* CpG methylation (β-value) between the non-paired normal and tumour tissue samples.**

| CpG Name | Mean diff(95% CI) | SE diff | p-value | Mean TP(95% CI) | SD mean TP | SEM TP | Mean NT(95% CI) | SD mean NT | SEM NT |
| --- | --- | --- | --- | --- | --- | --- | --- | --- | --- |
| cg17790129(CpG1) | -0.092(-0.135, -0.049) | 0.022 | 4.15E-05* | 0.831(0.529, 1.133) | 0.154 | 0.008 | 0.915(0.868, 0.962) | 0.024 | 0.004 |
| cg14205998(CpG2) | -0.120(-0.161, -0.079) | 0.021 | 1.19E-07* | 0.802(0.463, 1.141) | 0.173 | 0.009 | 0.928(0.879, 0.977) | 0.025 | 0.004 |
| cg04317854(CpG3) | -0.043(-0.074, -0.012) | 0.016 | 1.13E-02* | 0.792(0.561, 1.023) | 0.118 | 0.006 | 0.840(0.748, 0.932) | 0.047 | 0.007 |
| cg12922093(CpG4) | -0.177(-0.218, -0.136) | 0.021 | 1.45E-12* | 0.670(0.315, 1.025) | 0.181 | 0.009 | 0.842(0.779, 0.905) | 0.032 | 0.005 |
| cg17569154(CpG5) | -0.086(-0.139, -0.033) | 0.027 | 2.25E-03* | 0.552(0.178, 0.926) | 0.191 | 0.010 | 0.633(0.427, 0.839) | 0.105 | 0.016 |
| cg19260663(CpG6) | -0.099(-0.140, -0.058) | 0.021 | 5.54E-06* | 0.803(0.505, 1.101) | 0.152 | 0.008 | 0.902(0.849, 0.955) | 0.027 | 0.004 |
| cg09333471(CpG7) | 0.187(0.142, 0.232) | 0.023 | 3.39E-12* | 0.484(0.116, 0.852) | 0.188 | 0.01 | 0.308(0.224, 0.392) | 0.043 | 0.007 |
| cg00473134(CpG8) | 0.237(0.192, 0.282) | 0.023 | 3.63E-17* | 0.331(-0.049, 0.711) | 0.194 | 0.01 | 0.114(0.061, 0.167) | 0.027 | 0.004 |
| cg03995857(CpG9) | 0.392(0.337, 0.447) | 0.028 | 3.51E-24* | 0.498(0.026, 0.970) | 0.241 | 0.012 | 0.139(0.049, 0.229) | 0.046 | 0.007 |
| cg07320646(CpG10) | 0.345(0.267, 0.423) | 0.040 | 2.96E-14* | 0.368(-0.208, 0.944) | 0.294 | 0.015 | 0.024(0.004, 0.044) | 0.010 | 0.001 |
| cg07293520(CpG11) | 0.242(0.183, 0.301) | 0.030 | 2.20E-12* | 0.273(-0.207, 0.753) | 0.245 | 0.012 | 0.027(0.017, 0.037) | 0.005 | 0.001 |
| cg04770504(CpG12) | 0.293(0.242, 0.344) | 0.026 | 1.04E-20* | 0.320(-0.062, 0.702) | 0.195 | 0.010 | 0.021(0.013, 0.029) | 0.004 | 0.001 |
| cg24805239(CpG13) | 0.307(0.250, 0.364) | 0.029 | 1.19E-18* | 0.397(-0.040, 0.834) | 0.223 | 0.011 | 0.085(0.052, 0.118) | 0.017 | 0.003 |
| cg01733570(CpG14) | 0.111(0.054, 0.168) | 0.029 | 2.25E-04* | 0.590(0.186, 0.994) | 0.206 | 0.010 | 0.480(0.392, 0.568) | 0.045 | 0.007 |
| cg25723149(CpG15) | 0.107(0.054, 0.160) | 0.027 | 1.51E-04* | 0.631(0.245, 1.017) | 0.197 | 0.010 | 0.525(0.429, 0.621) | 0.049 | 0.007 |
| cg22804000(CpG16) | 0.105(0.052, 0.158) | 0.027 | 1.45E-04* | 0.565(0.185, 0.945) | 0.194 | 0.010 | 0.457(0.371, 0.543) | 0.044 | 0.007 |
| cg07504598(CpG17) | 0.111(0.068, 0.154) | 0.022 | 2.54E-06* | 0.677(0.340, 1.014) | 0.172 | 0.009 | 0.565(0.475, 0.655) | 0.046 | 0.007 |
| cg15037663(CpG18) | 0.067(0.018, 0.116) | 0.025 | 9.52E-03* | 0.627(0.292, 0.962) | 0.171 | 0.009 | 0.557(0.445, 0.669) | 0.057 | 0.009 |
| cg19706795(CpG19) | 0.072(0.033, 0.111) | 0.020 | 4.62E-04* | 0.711(0.450, 0.972) | 0.133 | 0.007 | 0.638(0.518, 0.758) | 0.061 | 0.009 |
| cg20764575(CpG20) | 0.029(0.002, 0.056) | 0.014 | 3.94E-02* | 0.805(0.629, 0.981) | 0.090 | 0.005 | 0.776(0.705, 0.847) | 0.036 | 0.005 |
| cg06301139(CpG21) | -0.091(-0.124, -0.058) | 0.017 | 7.05E-07* | 0.803(0.517, 1.089) | 0.146 | 0.007 | 0.902(0.835, 0.969) | 0.034 | 0.005 |
| cg26712096(CpG22) | -0.130(-0.185, -0.075) | 0.028 | 1.20E-05* | 0.457(0.061, 0.853) | 0.202 | 0.010 | 0.573(0.353, 0.793) | 0.112 | 0.017 |

TP = primary tumour, NT = normal tissue, Mean diff = difference in means (mean NT – mean TP), SE = standard error of difference of means, SD = standard deviation, SEM = standard error of the mean, CI = confidence interval, * represents significant results for which p-value < 0.05. Light shaded rows represent intragenic CpGs while dark shaded rows represent putative promoter CpGs.

**Supplementary Table 3. Differences in *GSDME* CpG methylation (β-value) between the paired normal and tumour tissue samples.**

| CpG Name | Mean diff(95% CI) | p-value |
| --- | --- | --- |
| cg17790129(CpG1) | 0.103(0.051, 0.155) | 2.48E-04* |
| cg14205998(CpG2) | 0.115(0.066, 0.164) | 2.60E-05* |
| cg04317854(CpG3) | 0.032(-0.008, 0.071) | 1.15E-01 |
| cg12922093(CpG4) | 0.178(0.128, 0.228) | 8.25E-09* |
| cg17569154(CpG5) | 0.105(0.035, 0.176) | 4.51E-03* |
| cg19260663(CpG6) | 0.100(0.052, 0.149) | 1.46E-04* |
| cg09333471(CpG7) | -0.199(-0.258, -0.141) | 2.04E-08* |
| cg00473134(CpG8) | -0.250(-0.304, -0.196) | 8.03E-12* |
| cg03995857(CpG9) | -0.420(-0.484, -0.356) | 1.66E-16* |
| cg07320646(CpG10) | -0.342(-0.437, -0.247) | 6.06E-09* |
| cg07293520(CpG11) | -0.237(-0.309, -0.164) | 5.70E-08* |
| cg04770504(CpG12) | -0.284(-0.346, -0.222) | 1.24E-11* |
| cg24805239(CpG13) | -0.300(-0.373, -0.227) | 2.16E-10* |
| cg01733570(CpG14) | -0.114(-0.187, -0.041) | 3.08E-03* |
| cg25723149(CpG15) | -0.107(-0.175, -0.040) | 2.54E-03* |
| cg22804000(CpG16) | -0.105(-0.170, -0.039) | 2.37E-03* |
| cg07504598(CpG17) | -0.112(-0.165, -0.060) | 9.62E-05* |
| cg15037663(CpG18) | -0.051(-0.116, 0.013) | 1.16E-01 |
| cg19706795(CpG19) | -0.062(-0.115, -0.008) | 2.53E-02* |
| cg20764575(CpG20) | -0.026(-0.065, 0.012) | 1.75E-01 |
| cg06301139(CpG21) | 0.088(0.048, 0.128) | 6.52E-05* |
| cg26712096(CpG22) | 0.155(0.079, 0.230) | 1.66E-04* |

CI = confidence interval, * represents significant results for which p-value < 0.05. Light shaded rows represent gene body CpGs while dark shaded rows represent putative promoter CpGs.

**Supplementary Table 4. Differences in *GSDME* CpG methylation (β-value) between the left-sided and right-sided CRC tissue groups.**

| CpG Names | Mean diff(95% CI) | SE diff | p-value | Mean L(95% CI) | SD mean L | SEM L | Mean R(95% CI) | SD mean R | SEM R |
| --- | --- | --- | --- | --- | --- | --- | --- | --- | --- |
| cg17790129(CpG1) | -0.033(-0.064, -0.002) | 0.016 | 4.21E-02* | 0.844(0.562, 1.126) | 0.144 | 0.010 | 0.817(0.496, 1.138) | 0.164 | 0.012 |
| cg14205998(CpG2) | -0.010(-0.045, 0.025) | 0.018 | 5.72E-01 | 0.806(0.471, 1.141) | 0.171 | 0.012 | 0.798(0.453, 1.143) | 0.176 | 0.013 |
| cg04317854(CpG3) | 0.024(0.000, 0.048) | 0.012 | 4.72E-02* | 0.781(0.528, 1.034) | 0.129 | 0.009 | 0.803(0.597, 1.009) | 0.105 | 0.008 |
| cg12922093(CpG4) | 0.001(-0.036, 0.038) | 0.019 | 9.59E-01 | 0.670(0.296, 1.044) | 0.191 | 0.013 | 0.671(0.336, 1.006) | 0.171 | 0.013 |
| cg17569154(CpG5) | -0.014(-0.053, 0.025) | 0.020 | 4.78E-01 | 0.561(0.181, 0.941) | 0.194 | 0.014 | 0.542(0.174, 0.910) | 0.188 | 0.014 |
| cg19260663(CpG6) | 0.008(-0.023, 0.039) | 0.016 | 6.33E-01 | 0.797(0.503, 1.091) | 0.150 | 0.011 | 0.809(0.505, 1.113) | 0.155 | 0.011 |
| cg09333471(CpG7) | 0.115(0.080, 0.150) | 0.018 | 1.14E-09* | 0.431(0.055, 0.807) | 0.192 | 0.014 | 0.541(0.216, 0.866) | 0.166 | 0.012 |
| cg00473134(CpG8) | 0.130(0.093, 0.167) | 0.019 | 1.98E-11* | 0.270(-0.085, 0.625) | 0.181 | 0.013 | 0.398(0.033, 0.763) | 0.186 | 0.014 |
| cg03995857(CpG9) | 0.161(0.116, 0.206) | 0.023 | 2.35E-11* | 0.422(-0.046, 0.890) | 0.239 | 0.017 | 0.580(0.159, 1.001) | 0.215 | 0.016 |
| cg07320646(CpG10) | 0.195(0.138, 0.252) | 0.029 | 4.63E-11* | 0.274(-0.285, 0.833) | 0.285 | 0.020 | 0.469(-0.058, 0.996) | 0.269 | 0.020 |
| cg07293520(CpG11) | 0.180(0.135, 0.225) | 0.023 | 1.66E-13* | 0.185(-0.227, 0.597) | 0.210 | 0.015 | 0.368(-0.112, 0.848) | 0.245 | 0.018 |
| cg04770504(CpG12) | 0.095(0.056, 0.134) | 0.020 | 1.55E-06* | 0.274(-0.122, 0.670) | 0.202 | 0.014 | 0.370(0.031, 0.709) | 0.173 | 0.013 |
| cg24805239(CpG13) | 0.150(0.107, 0.193) | 0.022 | 1.72E-11* | 0.324(-0.095, 0.743) | 0.214 | 0.015 | 0.476(0.074, 0.878) | 0.205 | 0.015 |
| cg01733570(CpG14) | 0.148(0.109, 0.187) | 0.020 | 1.21E-12* | 0.520(0.099, 0.941) | 0.215 | 0.015 | 0.666(0.341, 0.991) | 0.166 | 0.012 |
| cg25723149(CpG15) | 0.135(0.098, 0.172) | 0.019 | 1.29E-11* | 0.566(0.150, 0.982) | 0.212 | 0.015 | 0.700(0.404, 0.996) | 0.151 | 0.011 |
| cg22804000(CpG16) | 0.129(0.092, 0.166) | 0.019 | 3.59E-11* | 0.503(0.115, 0.891) | 0.198 | 0.014 | 0.632(0.309, 0.955) | 0.165 | 0.012 |
| cg07504598(CpG17) | 0.070(0.037, 0.103) | 0.017 | 7.27E-05* | 0.644(0.285, 1.003) | 0.183 | 0.013 | 0.712(0.414, 1.010) | 0.152 | 0.011 |
| cg15037663(CpG18) | 0.112(0.079, 0.145) | 0.017 | 1.03E-10* | 0.573(0.236, 0.910) | 0.172 | 0.012 | 0.686(0.394, 0.978) | 0.149 | 0.011 |
| cg19706795(CpG19) | 0.079(0.054, 0.104) | 0.013 | 5.83E-09* | 0.675(0.408, 0.942) | 0.136 | 0.010 | 0.751(0.522, 0.980) | 0.117 | 0.009 |
| cg20764575(CpG20) | 0.022(0.004, 0.040) | 0.009 | 1.68E-02* | 0.794(0.604, 0.984) | 0.097 | 0.007 | 0.817(0.660, 0.974) | 0.080 | 0.006 |
| cg06301139(CpG21) | 0.047(0.018, 0.076) | 0.015 | 1.74E-03* | 0.779(0.479, 1.079) | 0.153 | 0.011 | 0.830(0.571, 1.089) | 0.132 | 0.010 |
| cg26712096(CpG22) | 0.075(0.036, 0.114) | 0.020 | 2.62E-04* | 0.420(0.055, 0.785) | 0.186 | 0.013 | 0.496(0.082, 0.910) | 0.211 | 0.015 |

L = left-sided CRC, R = right-sided CRC, Mean diff = difference in means (mean R – mean L), SE = standard error of difference of means, SD = standard deviation, SEM = standard error of the mean, CI = confidence interval, * represents significant results for which p-value < 0.05. Light shaded rows represent intragenic CpGs while dark shaded rows represent putative promoter CpGs.

**Supplementary Table 5. Linear regression models of *GSDME* methylation vs RNAseq expression for the whole dataset, in addition to the Spearman correlation tests between methylation and RNAseq.**

| CpG Names | Slope | SE slope | p-value | Corr. coeff. | Corr. p-value |
| --- | --- | --- | --- | --- | --- |
| cg17790129(CpG1) | -0.274 | 0.581 | 6.38E-01 | 0.046 | 4.19E-01 |
| cg14205998(CpG2) | 0.307 | 0.539 | 5.69E-01 | 0.186 | 9.72E-04* |
| cg04317854(CpG3) | -1.921 | 0.860 | 2.62E-02* | -0.053 | 3.57E-01 |
| cg12922093(CpG4) | -0.236 | 0.515 | 6.47E-01 | 0.115 | 4.27E-02* |
| cg17569154(CpG5) | 0.417 | 0.498 | 4.03E-01 | 0.343 | 5.71E-10* |
| cg19260663(CpG6) | 1.527 | 0.634 | 1.66E-02* | 0.170 | 2.70E-03* |
| cg09333471(CpG7) | 0.032 | 0.750 | 9.66E-01 | -0.286 | 2.94E-07* |
| cg00473134(CpG8) | -0.812 | 0.671 | 2.27E-01 | -0.259 | 3.86E-06* |
| cg03995857(CpG9) | -1.517 | 0.603 | 1.24E-02* | -0.362 | 5.10E-11* |
| cg07320646(CpG10) | 0.476 | 0.658 | 4.70E-01 | -0.229 | 4.84E-05* |
| cg07293520(CpG11) | 0.086 | 0.695 | 9.02E-01 | -0.166 | 3.37E-03* |
| cg04770504(CpG12) | 0.592 | 0.965 | 5.40E-01 | -0.257 | 4.72E-06* |
| cg24805239(CpG13) | -0.295 | 0.946 | 7.56E-01 | -0.254 | 6.07E-06* |
| cg01733570(CpG14) | 0.808 | 0.801 | 3.14E-01 | -0.154 | 6.76E-03* |
| cg25723149(CpG15) | -0.482 | 1.075 | 6.54E-01 | -0.273 | 1.05E-06* |
| cg22804000(CpG16) | -0.615 | 0.938 | 5.12E-01 | -0.235 | 2.97E-05* |
| cg07504598(CpG17) | 0.432 | 0.619 | 4.86E-01 | -0.215 | 1.35E-04* |
| cg15037663(CpG18) | -0.228 | 0.749 | 7.61E-01 | -0.076 | 1.85E-01 |
| cg19706795(CpG19) | 0.255 | 0.992 | 7.97E-01 | -0.046 | 4.21E-01 |
| cg20764575(CpG20) | -2.437 | 1.139 | 3.32E-02* | -0.391 | 9.43E-13* |
| cg06301139(CpG21) | 0.775 | 0.673 | 2.51E-01 | 0.146 | 1.03E-02* |
| cg26712096(CpG22) | 2.871 | 0.527 | 1.07E-07* | 0.469 | 2.37E-18* |

SE slope = standard error of slope, Corr. Coeff. = Spearman’s correlation coefficient. * represents significant results for which p-value < 0.05. Light shaded rows represent intragenic CpGs while dark shaded rows represent putative promoter CpGs.

**Supplementary Table 6. Stepwise linear regression models of *GSDME* methylation vs RNAseq expression in the tumour vs. normal and left colon vs. right colon groups. For each of the four groups, CpG probes with a significant impact on RNAseq expression were selected through a stepwise linear regression, these were then used together in the final regression model for slope and p-value calculation. Probes that were non-significant were not included in the final model and are not shown.**

| Normal Tissue: R^2^=0.632, model p-value=1.38E-02 | | | |
| --- | --- | --- | --- |
| **Coefficients** | **Estimate slope** | **SE** | **p-value** |
| **cg09333471(CpG7)** | -10.118 | 7.425 | 1.96E-01 |
| **cg22804000(CpG16)** | 3.163 | 12.479 | 8.04E-01 |
| **cg15037663(CpG18)** | 3.182 | 7.991 | 6.97E-01 |
| **cg19706795(CpG19)** | 8.323 | 5.333 | 1.43E-01 |
| **cg20764575(CpG20)** | 21.671 | 9.210 | 3.50E-02* |
| Primary Tumuor: R^2^=0.396, model p-value< 2.2E-16 | | | |
| **cg04317854(CpG3)** | -2.018 | 0.739 | 6.66E-03* |
| **cg17569154(CpG5)** | 0.665 | 0.443 | 1.34E-01 |
| **cg19260663(CpG6)** | 1.639 | 0.560 | 3.68E-03* |
| **cg00473134(CpG8)** | -0.806 | 0.571 | 1.59E-01 |
| **cg03995857(CpG9)** | -1.485 | 0.504 | 3.46E-03* |
| **cg07320646(CpG10)** | 0.586 | 0.361 | 1.05E-01 |
| **cg20764575(CpG20)** | -2.337 | 0.928 | 1.23E-02* |
| **cg06301139(CpG21)** | 0.877 | 0.594 | 1.41E-01 |
| **cg26712096(CpG22)** | 2.934 | 0.450 | 2.94E-10* |
| Left Colon: R^2^=0.431, model p-value= 4.683E-11 | | | |
| **cg17569154(CpG5)** | 1.001 | 0.700 | 1.56E-01 |
| **cg03995857(CpG9)** | -1.681 | 0.640 | 9.99E-03* |
| **cg04770504(CpG12)** | 1.505 | 0.837 | 7.52E-02 |
| **cg15037663(CpG18)** | -1.631 | 0.851 | 5.81E-02 |
| **cg26712096(CpG22)** | 5.493 | 0.836 | 2.29E-09* |
| Right Colon: R^2^=0.415, model p-value< 2.2E-16 | | | |
| **cg17569154(CpG5)** | -1.626 | 1.022 | 1.14E-01 |
| **cg19260663(CpG6)** | 2.033 | 0.674 | 2.95E-03* |
| **cg03995857(CpG9)** | -2.400 | 0.588 | 6.69E-05* |
| **cg07320646(CpG10)** | 0.642 | 0.443 | 1.49E-01 |
| **cg20764575(CpG20)** | -3.839 | 1.269 | 2.84E-03* |
| **cg26712096(CpG22)** | 2.235 | 0.518 | 2.64E-05* |

Normal tissue N= 43, primary tumour N=389, left colon N=202, right colon N=187 SE = standard error of difference of means. * represents significant results for which p-value < 0.05. Light shaded rows represent intragenic CpGs while dark shaded rows represent putative promoter CpGs.

**Supplementary Table 7. Table showing the linear regression associations between CpG methylation and age, in addition to their Pearson’s correlation coefficients.**

| CpG Names | Slope Age | SE Slope | Age p-value | Correlation Coeff. | Correlation p-value |
| --- | --- | --- | --- | --- | --- |
| cg17790129(CpG1) | 3.27E-04 | 5.48E-04 | 5.51E-01 | 2.88E-02 | 5.51E-01 |
| cg14205998(CpG2) | 3.25E-05 | 6.22E-04 | 9.58E-01 | 2.52E-03 | 9.58E-01 |
| cg04317854(CpG3) | 5.61E-04 | 4.20E-04 | 1.83E-01 | 6.42E-02 | 1.83E-01 |
| cg12922093(CpG4) | 4.68E-05 | 6.63E-04 | 9.44E-01 | 3.40E-03 | 9.44E-01 |
| cg17569154(CpG5) | -4.10E-06 | 6.84E-04 | 9.95E-01 | -2.89E-04 | 9.95E-01 |
| cg19260663(CpG6) | 6.74E-04 | 5.43E-04 | 2.15E-01 | 5.98E-02 | 2.15E-01 |
| cg09333471(CpG7) | 2.92E-03 | 6.73E-04 | 1.79E-05* | 2.05E-01 | 1.79E-05* |
| cg00473134(CpG8) | 3.62E-03 | 6.97E-04 | 3.17E-07* | 2.43E-01 | 3.17E-07* |
| cg03995857(CpG9) | 3.37E-03 | 9.17E-04 | 2.66E-04* | 1.75E-01 | 2.66E-04* |
| cg07320646(CpG10) | 5.09E-03 | 1.07E-03 | 2.66E-06* | 2.24E-01 | 2.66E-06* |
| cg07293520(CpG11) | 4.36E-03 | 8.74E-04 | 8.75E-07* | 2.34E-01 | 8.75E-07* |
| cg04770504(CpG12) | 2.09E-03 | 7.50E-04 | 5.59E-03* | 1.33E-01 | 5.59E-03* |
| cg24805239(CpG13) | 3.48E-03 | 8.35E-04 | 3.66E-05* | 1.97E-01 | 3.66E-05* |
| cg01733570(CpG14) | 2.63E-03 | 7.22E-04 | 3.07E-04* | 1.73E-01 | 3.07E-04* |
| cg25723149(CpG15) | 2.88E-03 | 6.87E-04 | 3.30E-05* | 1.98E-01 | 3.30E-05* |
| cg22804000(CpG16) | 2.07E-03 | 6.82E-04 | 2.50E-03* | 1.45E-01 | 2.50E-03* |
| cg07504598(CpG17) | 1.65E-03 | 6.10E-04 | 7.14E-03* | 1.29E-01 | 7.14E-03* |
| cg15037663(CpG18) | 1.48E-03 | 6.03E-04 | 1.42E-02* | 1.18E-01 | 1.42E-02* |
| cg19706795(CpG19) | 5.82E-04 | 4.75E-04 | 2.21E-01 | 5.90E-02 | 2.21E-01 |
| cg20764575(CpG20) | 8.20E-04 | 3.15E-04 | 9.70E-03* | 1.24E-01 | 9.70E-03* |
| cg06301139(CpG21) | 1.99E-04 | 5.22E-04 | 7.04E-01 | 1.84E-02 | 7.04E-01 |
| cg26712096(CpG22) | -1.65E-03 | 7.24E-04 | 2.30E-02* | -1.09E-01 | 2.30E-02* |

SE Slope = standard error of slope, Correlation Coeff. = correlation coefficient (Pearson’s). * represents significant results for which p-value < 0.05. Light shaded rows represent intragenic CpGs while dark shaded rows represent putative promoter CpGs.

**Supplementary Table 8. Table showing the Cox-proportional hazard model statistics for the survival analysis.** Significance test results and effects sizes (hazard ratios) from the all models (age, tumour stage and methylation), for all 22 CpG islands.

| CpG Names | HR Age | SE HR Age | Age p-value | HR methylation | SE HR methylation | Methylation p-value |
| --- | --- | --- | --- | --- | --- | --- |
| Primary tumour dataset (N=340) | | | | | | |
| cg17790129(CpG1) | 1.038 | 0.012 | 1.12E-03* | 2.330 | 0.980 | 3.88E-01 |
| cg14205998(CpG2) | 1.041 | 0.012 | 7.20E-04* | 0.490 | 0.764 | 3.50E-01 |
| cg04317854(CpG3) | 1.039 | 0.012 | 9.90E-04* | 1.132 | 1.156 | 9.15E-01 |
| cg12922093(CpG4) | 1.040 | 0.012 | 8.10E-04* | 0.531 | 0.815 | 4.37E-01 |
| cg17569154(CpG5) | 1.038 | 0.012 | 1.17E-03* | 1.928 | 0.703 | 3.50E-01 |
| cg19260663(CpG6) | 1.040 | 0.012 | 9.30E-04* | 0.837 | 0.992 | 8.57E-01 |
| cg09333471(CpG7) | 1.038 | 0.012 | 1.78E-03* | 1.930 | 0.785 | 4.02E-01 |
| cg00473134(CpG8) | 1.039 | 0.012 | 1.38E-03* | 1.035 | 0.745 | 9.63E-01 |
| cg03995857(CpG9) | 1.037 | 0.012 | 1.92E-03* | 1.663 | 0.612 | 4.06E-01 |
| cg07320646(CpG10) | 1.037 | 0.012 | 2.09E-03* | 1.445 | 0.492 | 4.54E-01 |
| cg07293520(CpG11) | 1.035 | 0.012 | 3.97E-03* | 2.029 | 0.599 | 2.38E-01 |
| cg04770504(CpG12) | 1.038 | 0.012 | 1.62E-03* | 1.858 | 0.701 | 3.77E-01 |
| cg24805239(CpG13) | 1.037 | 0.012 | 2.26E-03* | 1.927 | 0.643 | 3.08E-01 |
| cg01733570(CpG14) | 1.036 | 0.012 | 2.59E-03* | 2.511 | 0.688 | 1.81E-01 |
| cg25723149(CpG15) | 1.036 | 0.012 | 2.83E-03* | 2.675 | 0.758 | 1.94E-01 |
| cg22804000(CpG16) | 1.037 | 0.012 | 2.12E-03* | 2.373 | 0.732 | 2.38E-01 |
| cg07504598(CpG17) | 1.038 | 0.012 | 1.56E-03* | 1.503 | 0.803 | 6.12E-01 |
| cg15037663(CpG18) | 1.038 | 0.012 | 1.30E-03* | 2.066 | 0.788 | 3.57E-01 |
| cg19706795(CpG19) | 1.039 | 0.012 | 1.00E-03* | 1.412 | 0.994 | 7.28E-01 |
| cg20764575(CpG20) | 1.039 | 0.012 | 1.09E-03* | 1.547 | 1.608 | 7.86E-01 |
| cg06301139(CpG21) | 1.040 | 0.012 | 8.60E-04* | 0.729 | 0.983 | 7.48E-01 |
| cg26712096(CpG22) | 1.039 | 0.011 | 8.10E-04* | 2.686 | 0.689 | 1.52E-01 |
| Left-sided dataset (N=176) | | | | | | |
| cg17790129(CpG1) | 1.043 | 0.0190 | 2.73E-02* | 1.501 | 1.841 | 8.25E-01 |
| cg14205998(CpG2) | 1.047 | 0.0193 | 1.86E-02* | 0.230 | 1.301 | 2.59E-01 |
| cg04317854(CpG3) | 1.043 | 0.0190 | 2.70E-02* | 1.003 | 1.793 | 9.99E-01 |
| cg12922093(CpG4) | 1.045 | 0.0191 | 2.24E-02* | 10.906 | 1.448 | 9.89E-02 |
| cg17569154(CpG5) | 1.042 | 0.0192 | 3.01E-02* | 2.705 | 1.113 | 3.71E-01 |
| cg19260663(CpG6) | 1.040 | 0.0192 | 4.27E-02* | 5.546 | 2.032 | 3.99E-01 |
| cg09333471(CpG7) | 1.043 | 0.0188 | 2.53E-02* | 0.925 | 1.200 | 9.48E-01 |
| cg00473134(CpG8) | 1.044 | 0.0190 | 2.31E-02* | 0.602 | 1.326 | 7.02E-01 |
| cg03995857(CpG9) | 1.043 | 0.0189 | 2.51E-02* | 0.909 | 0.924 | 9.18E-01 |
| cg07320646(CpG10) | 1.044 | 0.0187 | 1.98E-02* | 0.551 | 0.871 | 4.94E-01 |
| cg07293520(CpG11) | 1.044 | 0.0188 | 2.35E-02* | 0.696 | 1.150 | 7.52E-01 |
| cg04770504(CpG12) | 1.043 | 0.0191 | 2.73E-02* | 1.673 | 1.071 | 6.31E-01 |
| cg24805239(CpG13) | 1.043 | 0.0189 | 2.54E-02* | 0.962 | 1.012 | 9.69E-01 |
| cg01733570(CpG14) | 1.043 | 0.0190 | 2.61E-02* | 1.325 | 1.052 | 7.89E-01 |
| cg25723149(CpG15) | 1.042 | 0.0191 | 2.95E-02* | 2.308 | 1.077 | 4.37E-01 |
| cg22804000(CpG16) | 1.044 | 0.0193 | 2.72E-02* | 3.112 | 1.168 | 3.31E-01 |
| cg07504598(CpG17) | 1.042 | 0.0191 | 3.19E-02* | 2.005 | 1.216 | 5.67E-01 |
| cg15037663(CpG18) | 1.044 | 0.0191 | 2.43E-02* | 2.181 | 1.268 | 5.39E-01 |
| cg19706795(CpG19) | 1.043 | 0.0189 | 2.61E-02* | 0.827 | 1.638 | 9.08E-01 |
| cg20764575(CpG20) | 1.042 | 0.0187 | 2.74E-02* | 13.169 | 2.701 | 3.40E-01 |
| cg06301139(CpG21) | 1.043 | 0.0188 | 2.33E-02* | 16.363 | 1.910 | 1.43E-01 |
| cg26712096(CpG22) | 1.047 | 0.0199 | 2.18E-02* | 5.535 | 1.217 | 1.60E-02* |
| Right-sided dataset (N=164) | | | | | | |
| cg17790129(CpG1) | 1.020 | 0.014 | 1.63E-01 | 5.073 | 1.144 | 1.56E-01 |
| cg14205998(CpG2) | 1.024 | 0.015 | 1.09E-01 | 0.965 | 0.984 | 9.71E-01 |
| cg04317854(CpG3) | 1.024 | 0.014 | 1.04E-01 | 0.973 | 1.480 | 9.85E-01 |
| cg12922093(CpG4) | 1.027 | 0.015 | 6.93E-02 | 0.064 | 1.130 | 1.51E-02* |
| cg17569154(CpG5) | 1.023 | 0.014 | 1.20E-01 | 2.019 | 0.930 | 4.50E-01 |
| cg19260663(CpG6) | 1.025 | 0.015 | 9.33E-02 | 0.427 | 1.166 | 4.66E-01 |
| cg09333471(CpG7) | 1.024 | 0.015 | 1.13E-01 | 1.024 | 1.241 | 9.85E-01 |
| cg00473134(CpG8) | 1.028 | 0.015 | 7.09E-02 | 0.378 | 1.059 | 3.58E-01 |
| cg03995857(CpG9) | 1.024 | 0.015 | 1.07E-01 | 0.919 | 0.942 | 9.29E-01 |
| cg07320646(CpG10) | 1.023 | 0.015 | 1.28E-01 | 1.337 | 0.716 | 6.85E-01 |
| cg07293520(CpG11) | 1.022 | 0.015 | 1.63E-01 | 1.467 | 0.860 | 6.56E-01 |
| cg04770504(CpG12) | 1.024 | 0.015 | 1.04E-01 | 0.879 | 1.102 | 9.07E-01 |
| cg24805239(CpG13) | 1.023 | 0.015 | 1.21E-01 | 1.195 | 0.975 | 8.55E-01 |
| cg01733570(CpG14) | 1.021 | 0.015 | 1.60E-01 | 2.117 | 1.181 | 5.25E-01 |
| cg25723149(CpG15) | 1.024 | 0.015 | 1.09E-01 | 0.862 | 1.301 | 9.09E-01 |
| cg22804000(CpG16) | 1.025 | 0.015 | 9.36E-02 | 0.618 | 1.057 | 6.49E-01 |
| cg07504598(CpG17) | 1.024 | 0.015 | 1.03E-01 | 0.778 | 1.142 | 8.26E-01 |
| cg15037663(CpG18) | 1.024 | 0.015 | 9.83E-02 | 0.651 | 1.109 | 6.99E-01 |
| cg19706795(CpG19) | 1.024 | 0.015 | 1.01E-01 | 0.676 | 1.315 | 7.66E-01 |
| cg20764575(CpG20) | 1.026 | 0.015 | 7.95E-02 | 0.187 | 2.094 | 4.23E-01 |
| cg06301139(CpG21) | 1.027 | 0.015 | 7.75E-02 | 0.083 | 1.155 | 3.13E-02* |
| cg26712096(CpG22) | 1.024 | 0.014 | 9.85E-02 | 1.384 | 0.894 | 7.17E-01 |

HR Age = age hazard ration, SE HR Age = standard error of age hazard ratio, HR methylation = methylation hazard ratio, SE HR methylation = standard error of methylation hazard ratio. * represents significant results for which p-value < 0.05. Light shaded rows represent intragenic CpGs while dark shaded rows represent putative promoter CpGs.

**Supplementary Table 9. Table showing the likelihood ratio test p-values comparing the fit of the logistic regression model with both main effects, methylation and stage, and their interaction term, against the model with only the main effects.** The lack of significant p-values for either diseases stage or interaction between CpG methylation and disease stage for any of the CpGs shows the homogeneity of this marker across the different disease stages.

| CpG Names | p-value Stage | p-value Interaction (Methylation and Stage) |
| --- | --- | --- |
| cg17790129(CpG1) | 5.33E-01 | 7.01E-01 |
| cg14205998(CpG2) | 6.40E-01 | 2.92E-01 |
| cg04317854(CpG3) | 5.04E-01 | 4.42E-01 |
| cg12922093(CpG4) | 5.68E-01 | 4.81E-01 |
| cg17569154(CpG5) | 5.54E-01 | 7.71E-01 |
| cg19260663(CpG6) | 3.01E-01 | 2.18E-01 |
| cg09333471(CpG7) | 5.20E-01 | 8.57E-01 |
| cg00473134(CpG8) | 3.15E-01 | 5.90E-01 |
| cg03995857(CpG9) | 4.18E-01 | 7.24E-01 |
| cg07320646(CpG10) | 2.83E-01 | 6.48E-01 |
| cg07293520(CpG11) | 2.54E-01 | 6.18E-01 |
| cg04770504(CpG12) | 2.09E-01 | 5.79E-01 |
| cg24805239(CpG13) | 2.55E-01 | 5.25E-01 |
| cg01733570(CpG14) | 4.67E-01 | 7.32E-01 |
| cg25723149(CpG15) | 4.04E-01 | 6.92E-01 |
| cg22804000(CpG16) | 4.68E-01 | 8.27E-01 |
| cg07504598(CpG17) | 4.59E-01 | 7.42E-01 |
| cg15037663(CpG18) | 4.30E-01 | 6.85E-01 |
| cg19706795(CpG19) | 3.97E-01 | 7.08E-01 |
| cg20764575(CpG20) | 4.86E-01 | 8.41E-01 |
| cg06301139(CpG21) | 4.30E-01 | 6.39E-01 |
| cg26712096(CpG22) | 5.18E-01 | 3.78E-01 |

* represents significant results for which p-value < 0.05. Light shaded rows represent intragenic CpGs while dark shaded rows represent putative promoter CpGs.

**Supplementary Table 10. Table showing the prediction model’s area under the curve (AUC), for the different *GSDME* CpGs.**

| CpG Names | AUC |
| --- | --- |
| cg17790129(CpG1) | 0.644 |
| cg14205998(CpG2) | 0.791 |
| cg04317854(CpG3) | 0.585 |
| cg12922093(CpG4) | 0.776 |
| cg17569154(CpG5) | 0.646 |
| cg19260663(CpG6) | 0.713 |
| cg09333471(CpG7) | 0.793 |
| cg00473134(CpG8) | 0.800 |
| cg03995857(CpG9) | 0.859 |
| cg07320646(CpG10) | 0.831 |
| cg07293520(CpG11) | 0.830 |
| cg04770504(CpG12) | 0.870 |
| cg24805239(CpG13) | 0.839 |
| cg01733570(CpG14) | 0.708 |
| cg25723149(CpG15) | 0.714 |
| cg22804000(CpG16) | 0.712 |
| cg07504598(CpG17) | 0.756 |
| cg15037663(CpG18) | 0.650 |
| cg19706795(CpG19) | 0.701 |
| cg20764575(CpG20) | 0.699 |
| cg06301139(CpG21) | 0.652 |
| cg26712096(CpG22) | 0.707 |

AUC = area under the curve. Light shaded rows represent intragenic CpGs while dark shaded rows represent putative promoter CpGs.

**Supplementary Table 11. Table showing the mean methylation levels in the different groups for all 22 *GSDME* CpGs as well as their genomic locations.**

| CpG Names | Genomic Coordinate(Kb) | Mean NT Meth | Mean TP Meth | Mean L Meth | Mean R Meth |
| --- | --- | --- | --- | --- | --- |
| cg17790129(CpG1) | 24.738572 | 0.915 | 0.831 | 0.844 | 0.817 |
| cg14205998(CpG2) | 24.748668 | 0.928 | 0.802 | 0.806 | 0.798 |
| cg04317854(CpG3) | 24.762562 | 0.840 | 0.792 | 0.781 | 0.803 |
| cg12922093(CpG4) | 24.767644 | 0.842 | 0.670 | 0.670 | 0.671 |
| cg17569154(CpG5) | 24.781545 | 0.633 | 0.552 | 0.561 | 0.542 |
| cg19260663(CpG6) | 24.791121 | 0.902 | 0.803 | 0.797 | 0.809 |
| cg09333471(CpG7) | 24.796355 | 0.308 | 0.484 | 0.431 | 0.541 |
| cg00473134(CpG8) | 24.796494 | 0.114 | 0.331 | 0.270 | 0.398 |
| cg03995857(CpG9) | 24.796553 | 0.139 | 0.498 | 0.422 | 0.580 |
| cg07320646(CpG10) | 24.796981 | 0.024 | 0.368 | 0.274 | 0.469 |
| cg07293520(CpG11) | 24.797192 | 0.027 | 0.273 | 0.185 | 0.368 |
| cg04770504(CpG12) | 24.797363 | 0.021 | 0.320 | 0.274 | 0.370 |
| cg24805239(CpG13) | 24.797486 | 0.085 | 0.397 | 0.324 | 0.476 |
| cg01733570(CpG14) | 24.797656 | 0.480 | 0.590 | 0.520 | 0.666 |
| cg25723149(CpG15) | 24.79768 | 0.525 | 0.631 | 0.566 | 0.700 |
| cg22804000(CpG16) | 24.797691 | 0.457 | 0.565 | 0.503 | 0.632 |
| cg07504598(CpG17) | 24.797786 | 0.565 | 0.677 | 0.644 | 0.712 |
| cg15037663(CpG18) | 24.797835 | 0.557 | 0.627 | 0.573 | 0.686 |
| cg19706795(CpG19) | 24.797839 | 0.638 | 0.711 | 0.675 | 0.751 |
| cg20764575(CpG20) | 24.797884 | 0.776 | 0.805 | 0.794 | 0.817 |
| cg06301139(CpG21) | 24.798175 | 0.902 | 0.803 | 0.779 | 0.830 |
| cg26712096(CpG22) | 24.798855 | 0.573 | 0.457 | 0.420 | 0.496 |

Mean NT Meth = mean methylation in normal tissues, Mean TP Meth = mean methylation in tumour tissues, Mean L Meth = mean methylation in left-sided tissues, Mean R Meth = mean methylation in right-sided tissues. Light shaded rows represent intragenic CpGs while dark shaded rows represent putative promoter CpGs.

**
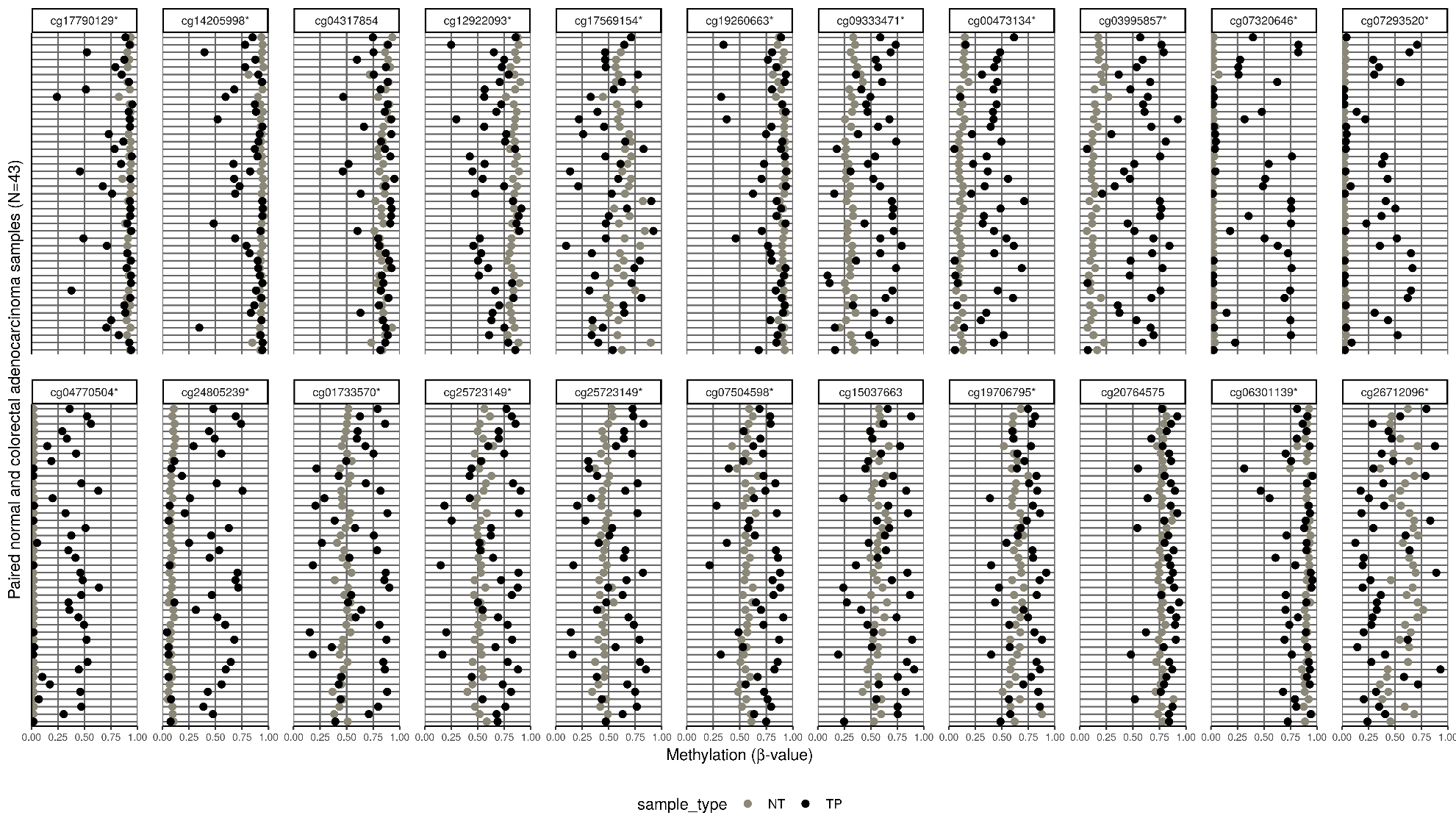
**

**Supplementary Figure 1: *GSDME* methylation in paired normal and colorectal adenocarcinoma samples (N=43).** For each CpG, methylation values of paired tissue samples are represented on the same horizontal line. A clear segregation in β-values can be observed between normal and tumour samples. Significantly different probes are marked CpGs in the putative promoter region seem be hypomethylated as compared to those in primary tumour tissues while the opposite holds for CpGs in the gene body. * represents significant results for which p-value < 0.05. Light shaded probes represent intragenic CpGs while dark shaded probes represent putative promoter CpGs.


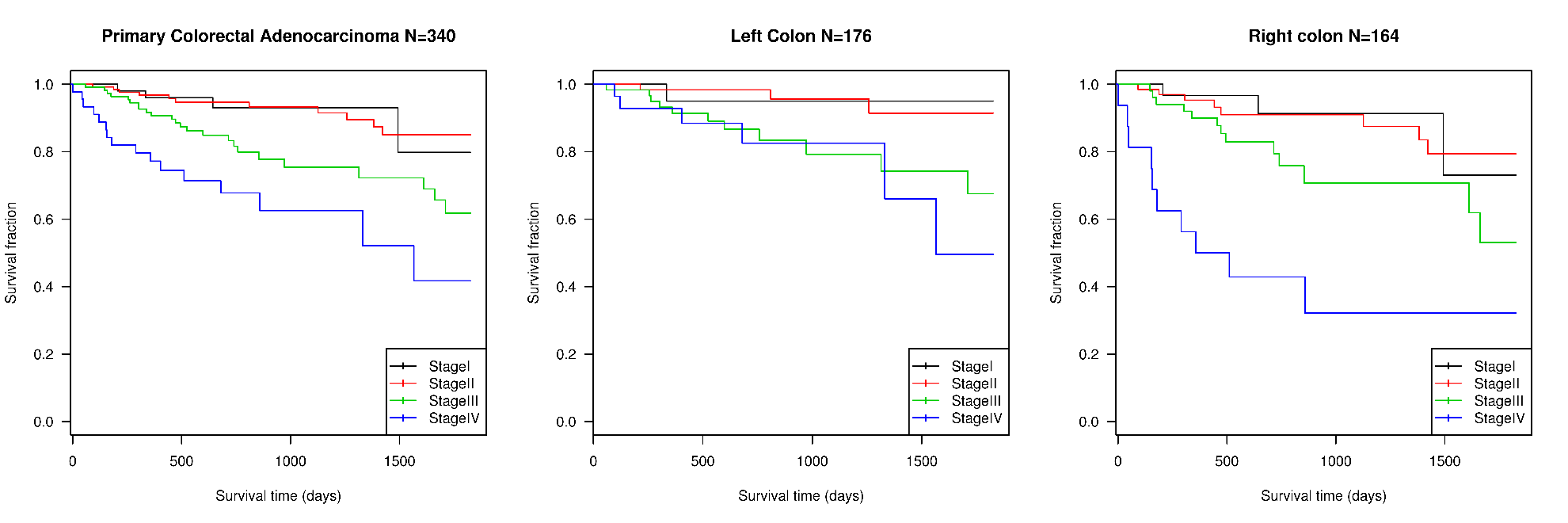


**Supplementary Figure 2. Kaplan Meier Survival plots (five-year) of the primary, left-sided and right-sided colorectal tumours, stratified by disease stage**. Methylation had no effect on survival, the only significant factor was disease stage.


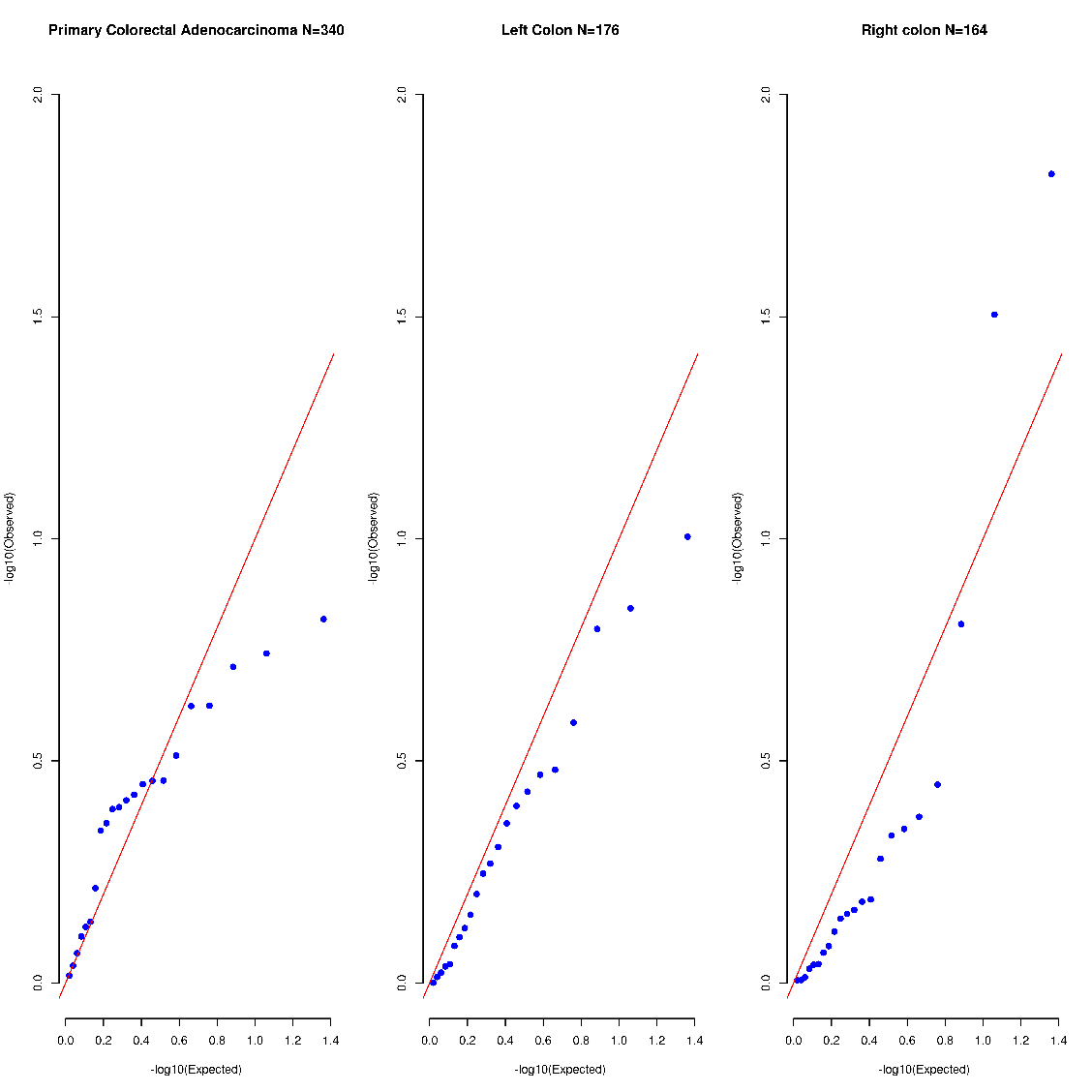


**Supplementary Figure 3. Q-Q plots p-values for the five-year survival analysis for primary, left-sided and right-sided colorectal tumours**. Under the null hypothesis, the CpG islands contain no information about survival, the p-values are expected to follow a uniform distribution between 0 and 1 (U(0,1)) . The diagonal line in the QQplot depicts that uniform distribution U(0,1). The dots on the QQplot represent the actual observed p-values. A deviation of the observed p-values toward more significant than expected under the U(0,1) distribution, is an indication. No significant enrichment in low p-values can be seen across the groups and hence no effect of methylation on survival can be concluded.


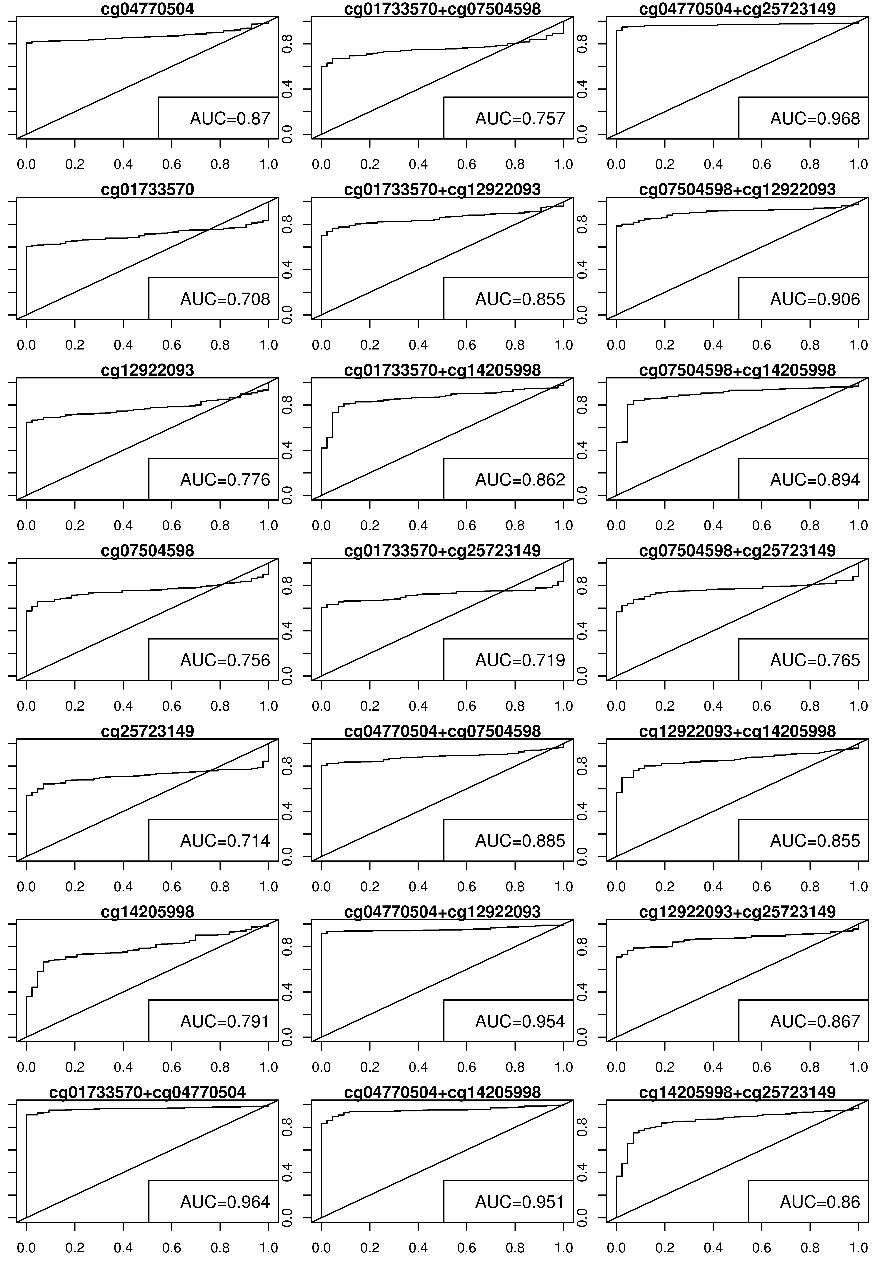


**Supplementary Figure 4. Receiver Operating Characteristic (ROC) curves of the six individual CpGs along with their pairwise combinations**. AUC = Area Under the Curve. The horizontal axis represents the false positive rate, while the vertical axis represents the true positive rate, the bisector line represents the AUC (0.5) at which no real distinction between the two outcomes is possible.


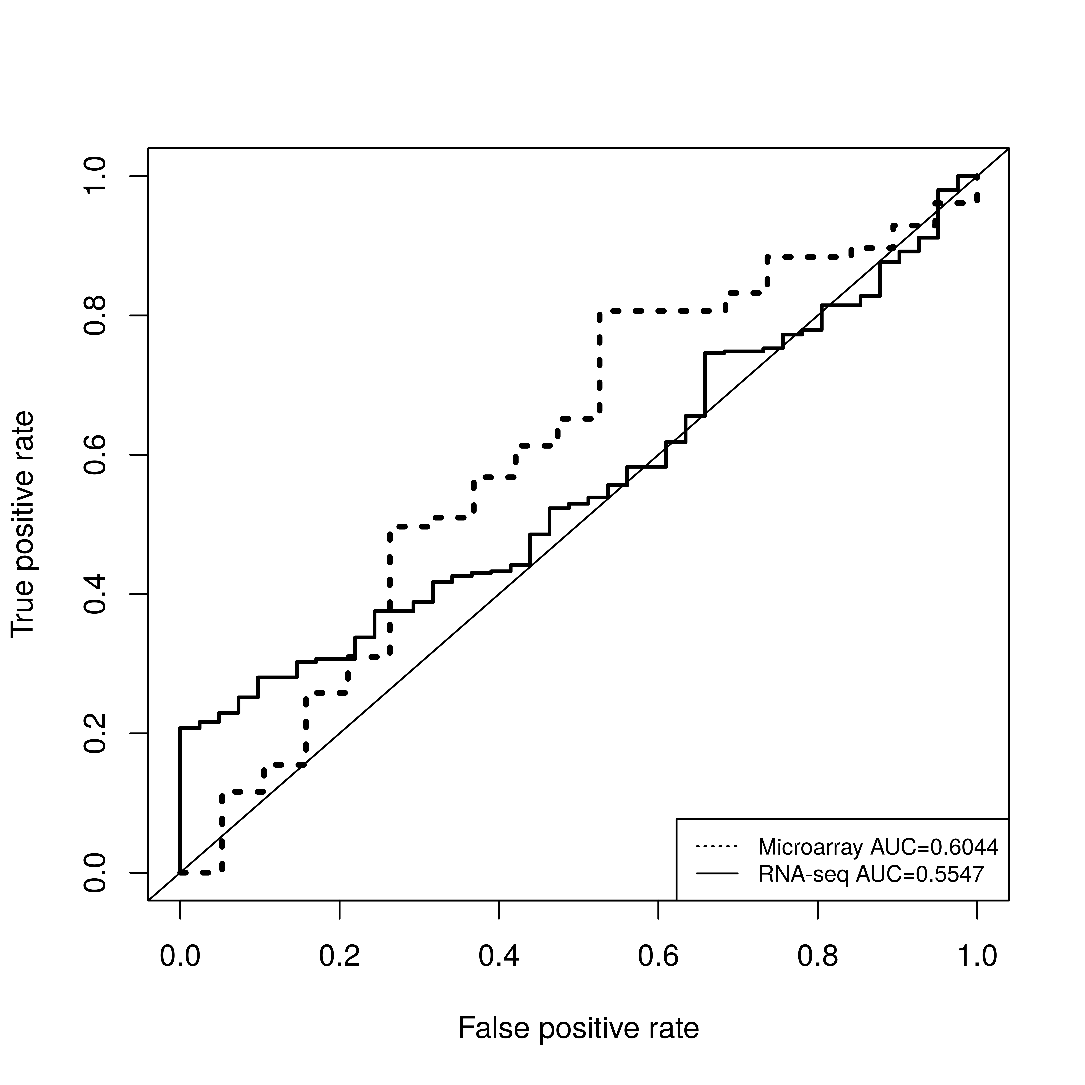


**Supplementary Figure 5. Receiver Operating Characteristic (ROC) curves of the expression datasets (RNAseq and microarray).** AUC = Area Under the Curve. The horizontal axis represents the false positive rate, while the vertical axis represents the true positive rate, the bisector line represents the AUC (0.5) at which no real distinction between the two outcomes is possible.


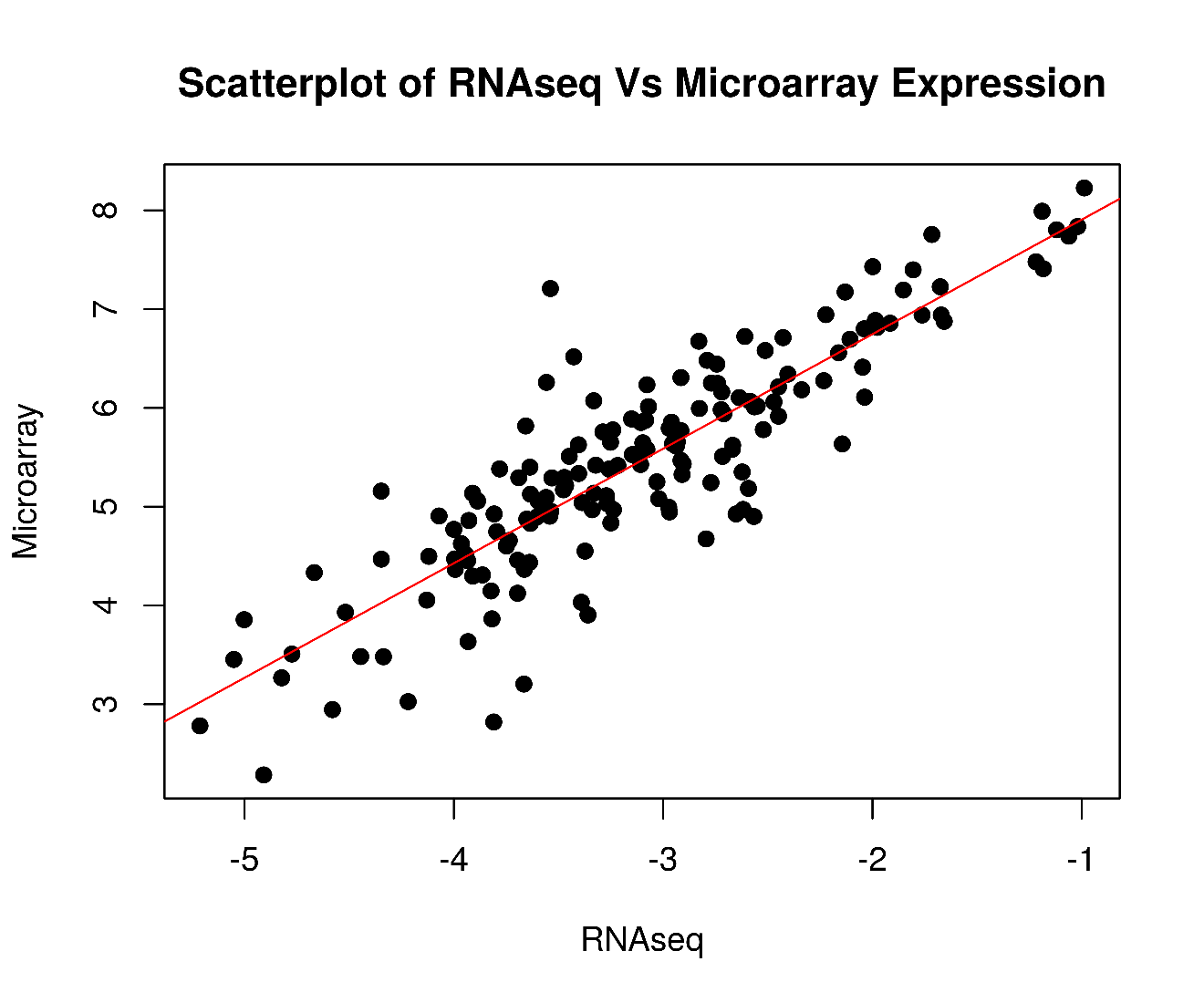


**Supplementary Figure 6. Scatterplot of RNAseq vs microarray expression data.** The two datasets are highly correlated with a Spearman’s coefficient of 0.89.

**R packages used:**

lme4 - Bates D, Mächler M, Bolker B, Walker S. Fitting Linear Mixed-Effects Models using lme4. *J Stat Softw*. 2014;67(1):51. doi:10.18637/jss.v067.i01.

Pbkrtest - Halekoh U, Højsgaard S. A Kenward-Roger Approximation and Parametric Bootstrap Methods for Tests in Linear Mixed Models - The R Package pbkrtest. *J Stat Softw*. 2014;59(9):1-32. doi:10.18637/jss.v059.i09.

Gap - Zhao JH. gap: Genetic Analysis Package. *J Stat Softw*. 2007;23(8):1-18.doi:http://dx.doi.org/10.18637/jss.v023.i08.

MASS - Venables WN (William N., Ripley BD, Venables WN (William N). *Modern Applied Statistics With S*. Vol 45. Fourth. New York: Springer; 2002. doi:10.1198/tech.2003.s33.

Corrplot - Wei T, Simko V. The corrplot package. *R Core Team*. 2016.

ROCR - Sing T, Sander O, Beerenwinkel N, Lengauer T. ROCR: Visualizing classifier performance in R. *Bioinformatics*. 2005;21(20):3940-3941. doi:10.1093/bioinformatics/bti623.

CvAUC - LeDell E, Petersen ML, Laan MJ van der. cvAUC: Cross-Validated Area Under the ROC Curve Confidence Intervals. 2013

**GEO external validation methylation datasets:**

GSE77718 – McInnes T, Zou D, Rao DS, Munro FM et al. Genome-wide methylation analysis identifies a core set of hypermethylated genes in CIMP-H colorectal cancer. *BMC Cancer.* Mar 28;17(1):228.

GSE42752 - Naumov VA, Generozov EV, Zaharjevskaya NB, Matushkina DS et al. Genome-scale analysis of DNA methylation in colorectal cancer using Infinium HumanMethylation450 BeadChips. *Epigenetics.* 2013;8(9):921-34.

GSE68060 - Conesa-Zamora P, García-Solano J, Turpin M del C, Sebastián-León P, Torres-Moreno D, Estrada E, Tuomisto A, Wilce J, Mäkinen M, Pérez-Guillermo M, Conesa A. Methylome profiling reveals functions and genes which are differentially methylated in serrated compared to conventional colorectal carcinoma. *Clinical Epigenetics*. 2015;7(1):101.
